# Endothelial dysfunction and proinflammatory state determine severe hematotoxicity and inferior outcome of CAR‐T therapy

**DOI:** 10.1002/hem3.70267

**Published:** 2025-12-08

**Authors:** Lukas Scheller, Xiang Zhou, Henry Loeffler‐Wirth, Markus Kreuz, Sofie‐Katrin Kadel, Sven Schimanski, Hannah Schulze, Anna Ruckdeschel, Florian Eisele, Verena Konetzki, Maria Jornet Culubret, Maximilian Merz, Julia Mersi, Johannes Waldschmidt, Sophia Danhof, Ulrike Köhl, K. Martin Kortüm, Leo Rasche, Hermann Einsele, Johannes Düll, Max S. Topp, Michael Hudecek, Kristin Reiche, Miriam Alb

**Affiliations:** ^1^ Medizinische Klinik und Poliklinik II Universitätsklinikum Würzburg Würzburg Germany; ^2^ Interdisziplinäres Zentrum für klinische Forschung (IZKF) Universitätsklinikum Würzburg Würzburg Germany; ^3^ Lehrstuhl für Zelluläre Immuntherapie, Medizinische Klinik und Poliklinik II Universitätsklinikum Würzburg Würzburg Germany; ^4^ Nationales Centrum für Tumorerkrankungen (NCT) Würzburg‐Erlangen‐Regensburg‐Augsburg (WERA) Würzburg Germany; ^5^ Interdisziplinäres Zentrum für Bioinformatik (IZBI) Universität Leipzig Leipzig Germany; ^6^ Fraunhofer‐Institut für Zelltherapie und Immunologie IZI Leipzig Germany; ^7^ Institut für klinische Immunologie Universität Leipzig Leipzig Germany; ^8^ Bayerisches Zentrum für Krebsforschung (BZKF) Leuchtturm Zelluläre Immuntherapie Würzburg Germany; ^9^ Fraunhofer‐Institut für Zelltherapie und Immunologie IZI, Leipzig, Außenstelle zelluläre Immuntherapie Würzburg Germany; ^10^ Center for Scalable Data Analytics and Artificial Intelligence Dresden/Leipzig Germany

## Abstract

Hematotoxicity and infections are the main drivers of non‐relapse mortality after chimeric antigen receptor (CAR)‐T therapy. Consequently, reliable predictive biomarkers are highly needed to improve risk assessment and optimize patient management. In this study, we applied the immune‐related adverse outcome pathway concept to delineate key events and risk factors of CAR‐T‐associated hematotoxicity. To identify predictive biomarkers, we performed flow cytometry and multiplex assays before and early after CAR‐T infusion on 78 patients (ide‐cel *n* = 31; axi‐cel *n* = 24; and cilta‐cel *n* = 23) undergoing CAR‐T therapy. Severe hematotoxicity was linked to endothelial dysfunction, as evidenced by reduced levels of ANG1, soluble selectins, and increased soluble VCAM‐1 (sVCAM‐1) early after CAR‐T infusion. Increased sVCAM‐1, reflecting endothelial dysfunction, elevated soluble IL‐2R (sIL‐2R), indicating a proinflammatory state, and high tumor burden before lymphodepletion were key risk factors for CAR‐T‐associated hematotoxicity. Patients with elevated sVCAM‐1 and sIL‐2R at baseline (pre‐lymphodepletion) exhibited significantly reduced overall survival (OS) (sVCAM‐1; P = 0.0009), prolonged Grade 4 neutropenia (sVCAM‐1; 12.1 vs. 6.0 days; P = 0.0016), more aplastic neutrophil recovery (5% vs. 30%; P = 0.007), and more severe infections (22.4% vs. 55%; P = 0.011). Baseline sIL‐2R and sVCAM‐1 demonstrated robust predictive value for prolonged neutropenia, severe infections, and mortality independently of key clinical variables such as the underlying disease and CAR‐T product. Integration of these markers improves existing models and can help to refine risk assessment and guide individualized patient management in CAR‐T therapy.

## Graphical abstract

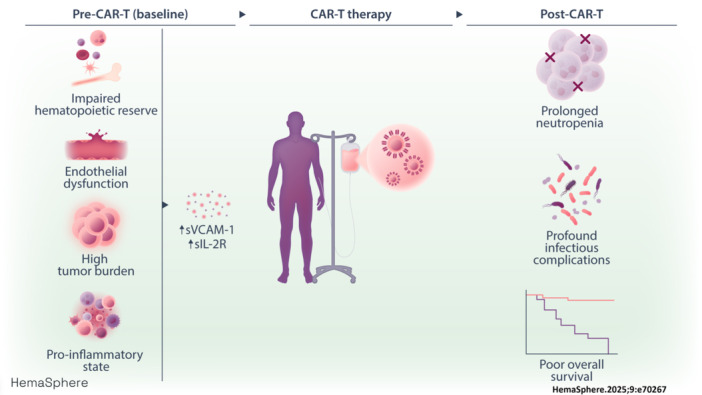

## INTRODUCTION

Hematotoxicity with prolonged cytopenia is one of the most relevant high‐grade toxicities after chimeric antigen receptor (CAR)‐T therapy in the real‐world setting.^1^, ^2^, ^3^ Prolonged cytopenia not only impairs the quality of life with the need for frequent medical consultations and transfusions but also potentiates the risk of severe infections.^4^ In line with this, a recent meta‐analysis of over 7600 patients treated with CAR‐T cells identified infections as the main driver of non‐relapse mortality irrespective of the disease entity and CAR‐T product.^5^ Therefore, biomarkers and predictive models for these adverse outcomes are highly needed to improve risk assessment and patient management.

CAR‐T‐associated hematotoxicity is the result of an interplay of multiple variables such as an impaired hematopoietic stem cell (HSC) reserve, endothelial dysfunction, and disruption of the bone marrow niche by local and systemic inflammation, as well as immune dysregulation.^6^ Prior myelotoxic treatments, natural ageing and interactions of the underlying disease with HSCs can lead to deregulation of hematopoiesis and impair the hematopoietic reserve. Within the bone marrow, HSCs are predominantly located adjacent to sinusoids, where a special niche composed of endothelial cells, perivascular stromal cells, and mesenchymal stromal cells promotes HSC maintenance by providing cytokines, forces, and cell‐mediated interactions.^7^ CAR‐T cells, when targeting tumor cells or non‐malignant antigen‐positive cells within the bone marrow, can induce cytokine release, local hyperinflammation, and endothelial dysfunction, disrupting the delicate balance within the HSC niche. Besides that, increased systemic levels of inflammatory mediators such as interferon‐γ (IFN‐γ), tumor necrosis factor‐α (TNF‐α), ferritin, and macrophage activation markers (e.g., MCP‐1) have been implicated with CAR‐T‐associated hematotoxicity.^8^, ^9^ Beyond directly impairing HSCs, increased inflammatory mediators suggest a pathomechanistic link to immune effector cell‐associated hemophagocytic lymphohistiocytosis‐like syndrome (IEC‐HS).^10^ This gives rise to the hypothesis that a high tumor burden, preexisting proinflammatory state, endothelial dysfunction, and impaired hematopoietic reserve are key determinants. Given the complexity of events leading to hematotoxicity, systematically delineating the process into key events is crucial to fill knowledge gaps and identify suitable models and biomarkers.

To this end, we employed the immune‐related adverse outcome pathway (irAOP) concept that enables the structuring of complex adverse outcomes like cytokine release syndrome (CRS) into defined and measurable key events.^11^, ^12^ This approach allows to capture the underlying pathomechanisms from a molecular and cellular level to the organism. In light of the combined CRS irAOP, we defined the binding of the CAR to its target as the molecular initiating event and (CAR‐) T and immune cell activation, cytokine release, local and systemic inflammation, endothelial dysfunction, and immune dysregulation as the subsequent key events contributing to the adverse outcome of hematotoxicity. Furthermore, we postulated that a baseline proinflammatory state, endothelial dysfunction, high tumor burden, and impaired hematopoietic reserve display key risk factors.

In this study, we applied the irAOP concept to identify novel biomarkers reflecting key events and risk factors of CAR‐T‐associated hematotoxicity to improve the prediction of adverse outcomes after CAR‐T therapy and thereby refine risk stratification.

## METHODS

### Study design

This study is part of the IMI2/EU project imSAVAR. To this end, patients treated with approved CAR‐T cells between November 2021 and August 2024 at Universitätsklinikum Würzburg were enrolled in a single‐center study. Patient characteristics, bridging therapies,^13^ laboratory and staging results, adverse events, and survival outcomes were collected with approval from the institutional review board and informed consent from the patients. All patients received standard fludarabine/cyclophosphamide lymphodepletion. Seventy‐eight patients treated with idecabtagene vicleucel (ide‐cel, *n* = 31), axicabtagene ciloleucel (axi‐cel, *n* = 24), and ciltacabtagene autoleucel (cilta‐cel, *n* = 23) completed sampling for all timepoints and were therefore included in the final analysis (Figure S1A,B). Patients treated with brexucabtagene autoleucel (brexu‐cel, *n* = 4) or tisagenlecleucel (tisa‐cel, *n* = 1) were excluded due to low numbers, which precluded meaningful statistical analysis. The study aimed for an observational period of at least 60 days, which was achieved in all but one patient, who was lost to follow‐up on Day 57. The median observation time was 369 days (ide‐cel 520 days, axi‐cel 408 days, and cilta‐cel 268 days). All procedures have been performed in accordance with the Declaration of Helsinki.

### Sampling and cell processing

Samples were collected before apheresis (BS1), at baseline, that is, before lymphodepletion (BS2), early after CAR‐T infusion (BS3, target Day 4 ± 1 for axi‐cel/ide‐cel and Day 9 ± 1 for cilta‐cel, to capture peak CAR‐T expansion and inflammation based on kinetic profiles of the respective product), and on Day 14 after CAR‐T infusion (BS4) (Figure S1A). Peripheral blood was collected in ethylenediaminetetraacetic acid (EDTA) anti‐coagulated and serum in clotting activator‐containing blood collection systems (Sarstedt, Nümbrecht, Germany). EDTA peripheral blood was processed freshly for flow cytometry. Serum was centrifuged at 1000 × *g* for 15 min at 8°C and stored at −80°C until further analysis.

### Grading and management of adverse events

Adverse events were classified according to the Common Terminology Criteria for Adverse Events (CTCAE) Version 5.0. CRS, immune effector cell‐associated neurotoxicity syndrome (ICANS), and immune effector cell‐associated hematotoxicity (ICAHT) were graded based on ASTCT and EHA/EBMT consensus grading.^14^, ^15^ Neutrophil recovery phenotypes were classified as quick, intermittent, and aplastic according to Rejeski et al.^3^ For calculation of cumulative cytopenia days, the intervening days between assessments were only counted if both values were below the respective threshold. CRS, ICANS, cytopenias, and infections were only analyzed from the day of CAR‐T infusion until disease progression or start of new treatments. Infections were defined as bacterial, viral, or fungal based on microbiology/virology test results, histopathologic data, or as a clinical syndrome of infection ± supportive imaging results. Fever alone after CAR‐T infusion without evidence of additional symptoms or microbiological or imaging evidence was not considered an infection. CRS/ICANS was managed in accordance with EHA/EBMT guidelines with early use of corticosteroids according to Topp et al.^16^ Dexamethasone was given at a standard dose of 10 mg. Growth factor support (granulocyte colony‐stimulating factor [G‐CSF], thrombopoietin [TPO] receptor agonists) and stem cell boosts were administered based on EHA/EBMT best practice recommendations.^15^ G‐CSF was used in patients with severe neutropenia only after resolution of CRS/ICANS. All patients received prophylactic acyclovir and co‐trimoxazole until CD4‐T‐cell count was >200 cells/μL and antifungal prophylaxis until neutrophil recovery. Patients with severe hypogammaglobulinemia (<4 g/L) received prophylactic intravenous immunoglobulins.

### Flow cytometry

Peripheral blood was freshly analyzed at the respective time points by flow cytometry including the following markers: CD45, CD3, CD4, CD8, CD19, CD16, CD56, HLA‐DR, CD14, CD11b, CD11c, CD33, and reagents to detect CD19‐ or BCMA‐CAR‐positive cells (Table S6). Samples were acquired on a MACSQuant10 (Miltenyi Biotec, Bergisch Gladbach, Germany) analyzer, and data were analyzed gated on live single cells with FlowJo (version 10.8.1) (Figure S11). Immune cell subsets were analyzed as frequencies of CD45^+^, live leukocytes. Additional information on flow cytometry is provided in the supplemental methods.

### Cytokine profiling

Cytokine profiling of 42 markers (Table S7) was performed on cryopreserved serum samples of all patients at the time point before lymphodepletion (BS2) and early after CAR‐T infusion (BS3). Analysis was performed employing three separate multiplex kits, analyzing 31, 9, and 2 analytes per run, to ensure optimal assay performance and minimize cross‐reactivity. Multiplex assays were purchased from ThermoFisher Scientific (Waltham, Massachusetts, USA) and BioTechne (Minneapolis, Minnesota, USA) and carried out according to the manufacturer's protocol. Assays were analyzed using a Luminex MAGPIX® system (Austin, Texas, USA) and ProcartaPlex Analysis Application (Version 3.1.1). Out‐of‐range cytokine values were set to the highest or lowest standard value. Marker selection was based on literature research to reflect defined key events and risk factors of CAR‐T‐associated hematotoxicity.

### Statistical analysis

Associations between continuous variables were assessed using Spearman's correlation (*r*). Binary variables were analyzed by logistic regression with likelihood ratio testing (*G*²). To adjust for multiple testing, *q*‐values were calculated, and a false discovery rate (FDR) cutoff < 0.1 was set.^17^ Progression‐free survival (PFS) and OS were calculated using the Kaplan–Meier method. Cox proportional hazards regression analysis was employed to evaluate the impact of covariates on survival outcomes. To assess the predictive value of biomarkers and scores, receiver operating characteristic (ROC) analyses were performed for each endpoint, with optimal cutoffs determined by maximizing the Youden index. The impact of identified markers on adverse outcomes was studied in univariate and multivariate logistic regression analysis. For logistic regression analysis, baseline concentrations of sIL‐2R and sVCAM‐1 were transformed using the natural logarithm as ln(*x* + 1) to stabilize variance, reduce the influence of extreme values, and improve model interpretability. Statistical significance between groups was assessed by the nonparametric Wilcoxon–Mann–Whitney *U* test for comparison of continuous variables and Fisher's exact test to compare categorical variables. GraphPad Prism (version 9.4.1) and R statistical software (version 4.3.1) were used for statistical analysis. The datasets generated in this study are available from the corresponding author on reasonable request.

## RESULTS

### Severe neutropenia and infections are the most frequent adverse events after CAR‐T therapy and lead to poor outcomes

Among the 78 patients analyzed, 54 had relapsed/refractory multiple myeloma (MM) treated with ide‐cel (*n* = 31) or cilta‐cel (*n* = 23), while 24 had diffuse large B‐cell lymphoma (DLBCL) treated with axi‐cel. The patient characteristics are summarized in Table 1 with detailed description including bridging therapies, incidence of adverse events, and causes of deaths (Tables S1–S3) in the supplements. Overall, our cohort included patients with high‐risk profiles, high tumor burden, and intensive pretreatments reflecting a typical representation of patients currently treated with the most commonly used approved CAR‐T products for MM and DLBCL patients in the real‐world setting.

**Table 1:** Baseline patient characteristics.

Characteristics	All patients (*n* = 78)
Male—no. (%)	48 (62%)
Female—no. (%)	30 (38%)
Age—median (range)	64 (22–79)
Tumor entity—no. (%)	
Multiple myeloma	54 (69%)
DLBCL	24 (31%)
**Disease stage/risk profile**	
MM	
R‐ISS III—no. (%)	7 (13%)
High‐risk cytogenetics (IMWG consensus criteria 2024—no. (%))	21 (39%)
Extramedullary disease—no. (%)	16 (30%)
Elevated LDH—no. (%)	9 (17%)
DLBCL	
Ann Arbor ≥ 3—no. (%)	17 (71%)
IPI ≥ 3—no. (%)	10 (42%)
Bulk or elevated LDH—no. (%)	14 (58%)
**Prior therapies—MM**	
Prior therapy lines—median (range)	5 (2–10)
Lenalidomide exposed—no. (%)	52 (96%)
Pomalidomide exposed—no. (%)	46 (85%)
Bortezomib exposed—no. (%)	52 (96%)
Carfilzomib exposed—no. (%)	49 (91%)
Daratumumab exposed—no. (%)	54 (100%)
Triple‐class refractory—no. (%)	40 (74%)
Penta‐class refractory—no. (%)	10 (19%)
Prior autologous SCT—no. (%)	53 (98%)
Prior allogeneic SCT—no. (%)	7 (13%)
**Prior therapies—DLBCL**	
Prior therapy lines—median (range)	2 (1–5)
Primary refractory disease—no. (%)	12 (50%)
Refractory to second or subsequent lines—no. (%)	12 (50%)
Early relapse (<12 months) after first‐line treatment —no. (%)	9 (38%)
Prior autologous SCT lymphoma—no. (%)	5 (21%)
**CAR‐T products**	78
Idecabtagene vicleucel (ide‐cel)—no. (%)	31 (40%)
Axicabtagene ciloleucel (axi‐cel)—no. (%)	24 (31%)
Ciltacabtagene autoleucel (cilta‐cel)—no. (%)	23 (30%)

Abbreviations: CAR, chimeric antigen receptor; DLBCL, diffuse large B‐cell lymphoma; IMWG, International Myeloma Working Group; IPI, International Prognostic Index; LDH, lactate dehydrogenase; MM, multiple myeloma; R‐ISS, Revised International Staging System; SCT, stem cell transplantation.

**Figure 1:**
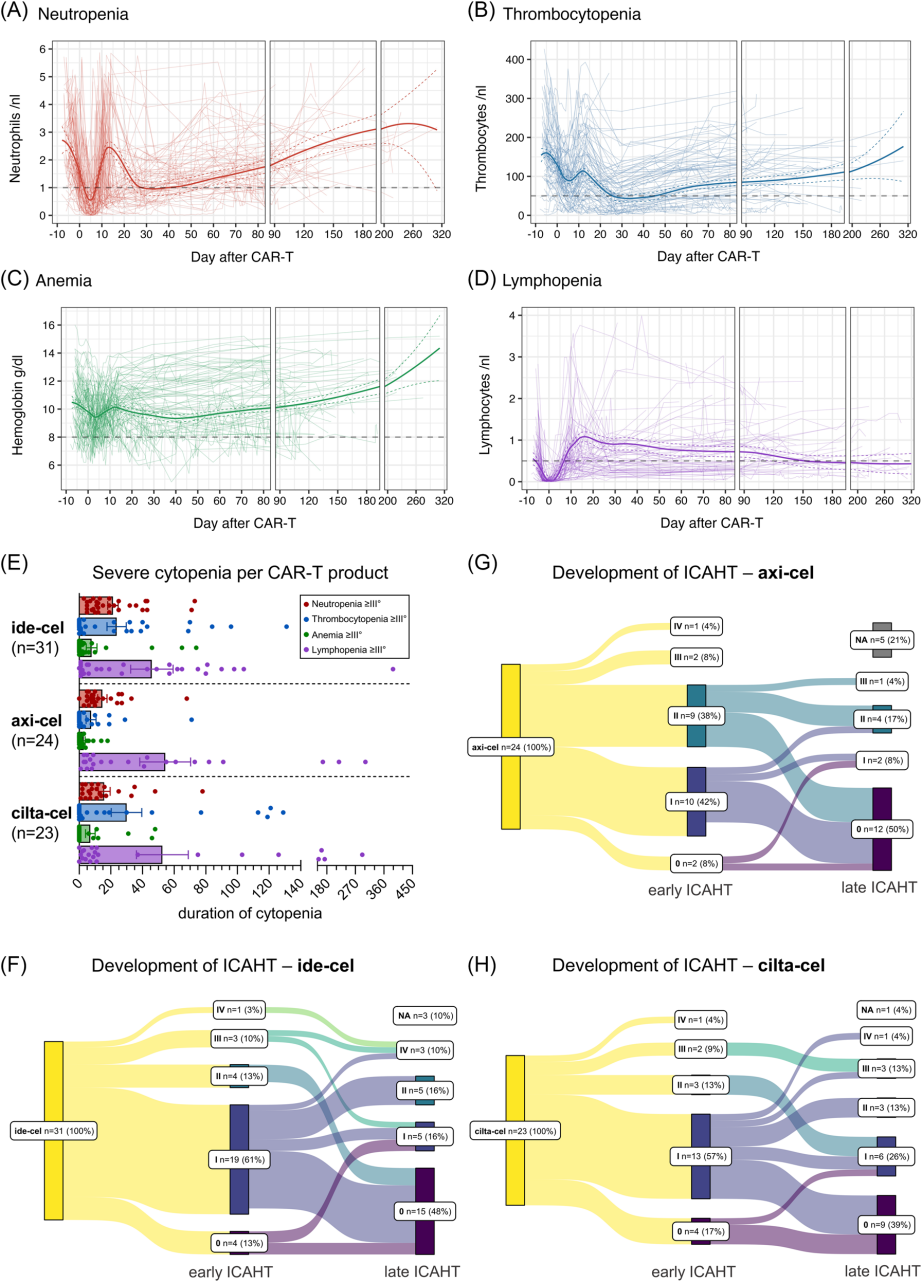
**Incidence and severity of hematologic toxicities after chimeric antigen receptor (CAR)‐T**. **(A–D)** Blood counts of neutrophils **(A)**, thrombocytes **(B)**, hemoglobin level **(C)**, and lymphocytes **(D)** before start of lymphodepletion and after CAR‐T infusion shown as spaghetti plots depicting the individual course of every single patient (thin lines) as well as the best‐fit line from local polynomal regression (bold line) and its 95% confidence interval (dashed colored lines). The dashed gray lines show the border of respective Grade 3 cytopenias. **(E)** Mean cumulative duration of Grade ≥3 neutropenia, thrombocytopenia, anemia, and lymphopenia for ide‐cel, axi‐cel, and cilta‐cel patients. Whiskers indicate standard error of the mean (SEM). Statistical significance of the duration of cytopenias between the different CAR‐T products was tested by Kruskal–Wallis and Dunn's multiple comparison test. **(F–H)** Sankey diagrams showing the time course from early immune effector cell‐associated hematotoxicity (ICAHT) grade to late ICAHT grade for ide‐cel **(F)**, axi‐cel **(G)**, and cilta‐cel **(H)**. NA = not available. Statistical significance of the frequency of early and late ICAHT between the different CAR‐T products was tested by Fisher's exact test. Of the three ide‐cel patients not available (NA) for late ICAHT classification, two had progressive disease, and one was lost to follow‐up on Day 82 with only one visit after Day 30. Of the five axi‐cel patients not available (NA) for late ICAHT classification, four had progressive disease, and one patient deceased due to severe infection (early ICAHT Grade 3). The one cilta‐cel patient not available (NA) for late ICAHT classification deceased of intracranial hemorrhage with only one visit after Day 30.

Among the 78 patients included in this study, high‐grade CRS (Grade ≥3) occurred in only one patient (<1%). In contrast, ICANS of Grade ≥3 was observed in seven patients, all within the DLBCL cohort, including six Grade 3 and one Grade 4 events (Table 2). Infections were recorded in 52 patients (67%), with a higher rate following BCMA‐ than CD19‐directed CAR‐T. Severe infections (≥Grade 3) were reported in 24 patients (31%). Nearly all patients developed Grade ≥3 neutropenia (99%) or lymphopenia (94%), while Grade ≥3 anemia and thrombocytopenia were observed in 46% and 59% of patients, respectively (Figure 1A–D). In addition, 40% developed prolonged Grade ≥3 neutropenia, and 14% developed prolonged Grade ≥4 neutropenia of more than 14 days. Twenty‐six patients (33%) developed prolonged Grade ≥3 thrombocytopenia of more than 14 days. The mean cumulative days of Grade ≥3 thrombocytopenia, neutropenia, and lymphopenia were 20.6 (range 0–131), 17.7 (range 0–78), and 50.5 (range 0–389).

**Table 2:** Incidence and severity of adverse events after chimeric antigen receptor (CAR)‐T.

Adverse events	
**CRS any grade—no. (%)** a	71 (91%)
I°	33 (42%)
II°	37 (47%)
III°	1 (1%)
IV°	0
V°	0
Median onset day (range)	1 (0–10)
Median duration (range)	4 (1–12)
CRS ≥ 3 by disease—no. (%)	
MM/ide‐cel or cilta‐cel	0 (0%)
DLBCL/axi‐cel	1 (4%)
**ICANS any grade—no. (%)** a	16 (21%)
I°	7 (9%)
II°	2 (3%)
III°	6 (8%)
IV°	1 (1%)
V°	0
Median onset day (range)	6 (1–9)
Median duration (range)	8 (1–12)
ICANS ≥ 3 by disease—no. (%)	
MM/ide‐cel or cilta‐cel	0
DLBCL/axi‐cel	7 (29%)
**Other neurotoxicities—cilta‐cel**	
Cranial nerve palsy	2 (9%)
MNT	1 (4%)
**Medication—no. (%)**	
Tocilizumab received	63 (81%)
Corticosteroids	64 (82%)
**Infections any grade—no. (%)** b	52 (67%)
0°	26 (33%)
I°	7 (9%)
II°	21 (27%)
III°	15 (19%)
IV°	5 (6%)
V°	4 (5%)
**Cause of infection**	
Viral	29 (37%)
Bacterial	20 (26%)
Fungal	3 (4%)
Unknown	8 (10%)
**Cytopenia any grade—no. (%)** b	78 (100%)
Anemia CTCAE ≥ 3	36 (46%)
Thrombocytopenia CTCAE ≥ 3	46 (59%)
Thrombocytopenia CTCAE ≥ 3 for ≥14 days	26 (33%)
Mean cumulative days of thrombocytopenia < 50 G/L (range)	20.6 (0–131)
Transfusion required (pRBC or platelets)	38 (49%)
Lymphopenia CTCAE ≥ 3	73 (94%)
Lymphopenia CTCAE ≥ 3 for ≥14 days	38 (49%)
Mean cumulative days of lymphopenia < 500/µL (range)	50.5 (0–389)
Neutropenia CTCAE ≥ 3	77 (99%)
Neutropenia CTCAE ≥ 3 for ≥14 days	31 (40%)
Mean cumulative days of neutropenia < 1000/µL (range)	17.7 (0–78)
Neutropenia CTCAE ≥ 4	70 (90%)
Neutropenia CTCAE ≥ 4 for ≥14 days	11 (14%)
Neutropenia CTCAE ≥ 4 for ≥21 days	6 (8%)
Mean cumulative days of neutropenia < 500/µL (range)	7.6 (0–44)
G‐CSF support d1–14	67 (86%)
G‐CSF support after d14	30 (39%)
TPO receptor agonists received	5 (6%)
Autologous stem cell boost	2 (3%)
Allogeneic stem cell transplant	1 (1%)
CAR‐HEMATOTOX high^3^ (≥2)	26 (33%)
**Neutrophil recovery type** c	
Quick	33 (42%)
Intermittent	36 (46%)
Aplastic	9 (12%)
**Secondary malignancies**	
Myelodysplastic syndrome (MDS)	2 (3%)
Acute myeloid leukemia (AML)	2 (3%)
**Other adverse events**	
Immune effector cell‐associated hemophagocytic lymphohistiocytosis‐like syndrome (IEC‐HS)	0
Cardiovascular events	0

Abbreviations: ANC, absolute neutrophil count; CRS, cytokine release syndrome; ASTCT, American Society for Transplantation and Cellular Therapy; CTCAE, Common Terminology Criteria for Adverse Events; DLBCL, diffuse large B‐cell lymphoma; G‐CSF, granulocyte colony‐stimulating factor; ICANS, immune effector cell‐associated neurotoxicity syndrome; MM, multiple myeloma; MNT, movement and neurocognitive treatment‐emergent adverse events; pRBC, packed red blood cells; TPO, thrombopoietin.

^a^ As per ASTCT consensus criteria.

^b^ As per CTCAE Version 5.0 criteria.

^c^ Quick: sustained neutrophil recovery without second dip below ANC < 1000/µL; intermittent: neutrophil recovery (ANC > 1500 cells per µL) followed by a second dip with ANC < 1000/µL after Day 21; aplastic: continuous severe neutropenia (ANC < 500/µL) for ≥14 days.

The relevance of cytopenias post CAR‐T is further highlighted by the number of patients requiring transfusions (49%) or G‐CSF support before (86%) or later than day 14 (39%) after CAR‐T infusion (Table 2). Two patients received autologous and one patient allogeneic stem cell boost due to prolonged aplasia. Furthermore, prolonged Grade ≥4 neutropenia was found to be significantly associated with death, life‐threatening infections, and poor peak CAR‐T expansion after CAR‐T therapy (Figure S1C–E). Among all cytopenias, prolonged neutropenia had the highest degree of overlap with other cytopenias, most frequently co‐occurring with lymphopenia, followed by thrombocytopenia (Figure S1F).

Looking at the different CAR‐T products, MM patients treated with ide‐cel or cilta‐cel trended to develop more prolonged thrombocytopenias; however, no significant differences in the duration of any cytopenia could be observed compared to DLBCL patients treated with axi‐cel (Figure 1E; Table S2). Additionally, whereas patients treated with ide‐cel, axi‐cel, and cilta‐cel had comparable early hematotoxicity rates, ide‐cel and cilta‐cel patients trended to develop more severe late hematotoxicity (late ICAHT ≥ 3 9.7% ide‐cel vs. 17.4% cilta‐cel vs. 4.2% axi‐cel, n.s.) (Figure 1F–H). However, it remains unclear whether these differences are attributable to disease‐specific factors or the CAR‐T product itself. In univariate analysis, neither cilta‐cel nor axi‐cel was significantly associated with an increased risk of prolonged neutropenia ≥ 7 days, severe infection (≥Grade 3), or death when compared to ide‐cel (Table S4). Of note, besides duration of Grade ≥3 lymphopenia, there was no significant difference in the duration of other severe cytopenias between responders and non‐responders (Figure S1G).

In summary, hematotoxicity with prolonged cytopenias and associated infections were the most relevant adverse events after CAR‐T therapy, with prolonged neutropenia significantly linked to poor outcomes.

### Severe hematotoxicity after CAR‐T therapy is associated with endothelial dysfunction

**Figure 2:**
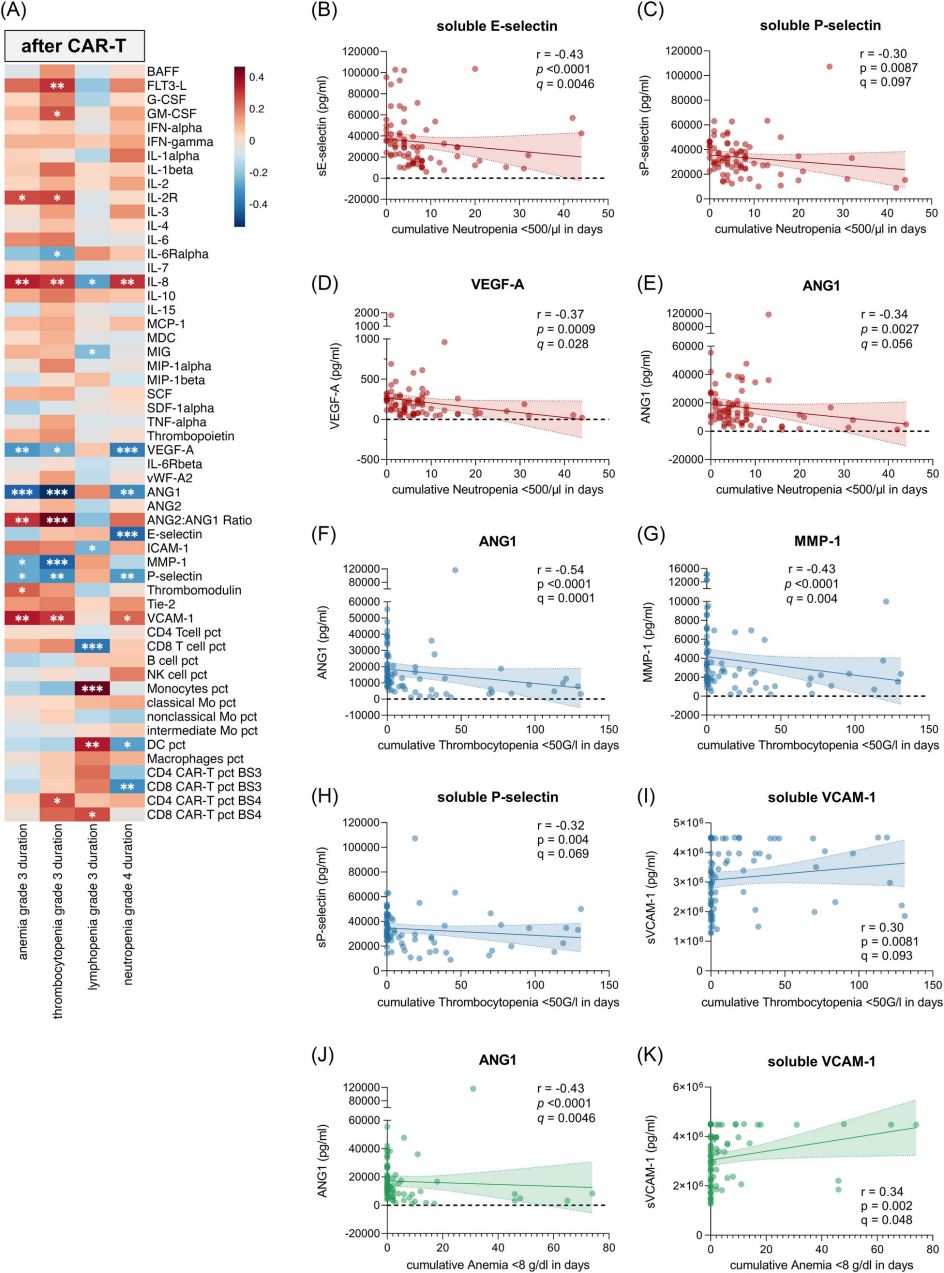
**Post‐infusion factors associated with severe hematotoxicity after chimeric antigen receptor (CAR)‐T**. **(A)** Spearman correlation analysis of the influence of cytokines and flow cytometry markers early after CAR‐T infusion (Days 3–10; BS3) and cumulative Grade ≥3 anemia, Grade ≥3 thrombocytopenia, Grade ≥3 lymphopenia, and Grade ≥4 neutropenia. The Spearman correlation coefficient (*r* = scalebar) and the respective P values are depicted in the heatmap with red indicating positive correlation and blue indicating negative correlation (*P < 0.05, **P < 0.01, and ***P < 0.001). **(B–H)** Univariate analysis of the influence of soluble E‐selectin **(B)**, soluble P‐selectin **(C)**, VEGF‐A **(D)**, and ANG1 **(E)** on prolonged neutropenia (red), ANG1 **(F)**, MMP‐1 **(G)**, soluble P‐selectin **(H)**, and soluble VCAM‐1 **(I)** on prolonged thrombocytopenia (blue) as well as ANG1 **(J)** and soluble VCAM‐1 **(K)** on prolonged anemia (green) after CAR‐T therapy, each depicted in comparison to cumulative duration. Best‐fit line (bold line) and 95% confidence intervals (dashed line) were calculated by simple linear regression. The Spearman correlation coefficient (*r*) and respective P‐ and *q*‐values (after adjustment for multiple testing) are depicted for each marker. Shown are selected markers that fulfilled a *q*‐value cutoff of <0.1. DC, dendritic cells; Mo, monocytes; pct, percentage.

To study markers reflecting key events and risk factors of CAR‐T‐associated hematotoxicity, we characterized patients undergoing CAR‐T therapy by flow cytometry and cytokine analysis (Figure S1A). First, we analyzed the association between factors detected early after CAR‐T infusion (Days 3–10, BS3) and the occurrence of prolonged cytopenias. We found several correlating markers (Figure 2A), of which 23—predominantly associated with endothelial dysfunction—remained significant after adjustment for multiple testing (*q* < 0.1) (Figures 2B–K; S2A–F). More specifically, reduced ANG1 and concomitantly increased ANG2:ANG1 ratio correlated with prolonged anemia, thrombocytopenia, and neutropenia early after CAR‐T (Figure 2E, F, and J). In addition, other endothelial markers such as reduced soluble P‐selectin significantly correlated with prolonged thrombocytopenia and neutropenia (Figure 2C,H), reduced VEGF‐A with prolonged neutropenia and anemia (Figures 2D; S2F), and increased soluble VCAM‐1 (sVCAM‐1) with prolonged thrombocytopenia and anemia (Figure 2I,K). Finally, reduced soluble E‐selectin levels showed the strongest but sole association with prolonged neutropenia, and reduced MMP‐1 was almost exclusively associated with prolonged thrombocytopenia after CAR‐T (Figure 2B,G).

Taken together, these findings highlight endothelial dysfunction as a key event of severe hematotoxicity after CAR‐T therapy. Additionally, consistent with previous reports,^18^, ^19^ we observed that CRS and ICANS were also significantly associated with markers of endothelial dysfunction, such as sVCAM‐1, ANG2, and vWF‐A2, further supporting the central role of endothelial dysfunction in CAR‐T–related toxicities (Figure S3A).

### Endothelial dysfunction, a proinflammatory state, and high tumor burden at baseline are key risk factors of CAR‐T‐associated hematotoxicity

**Figure 3:**
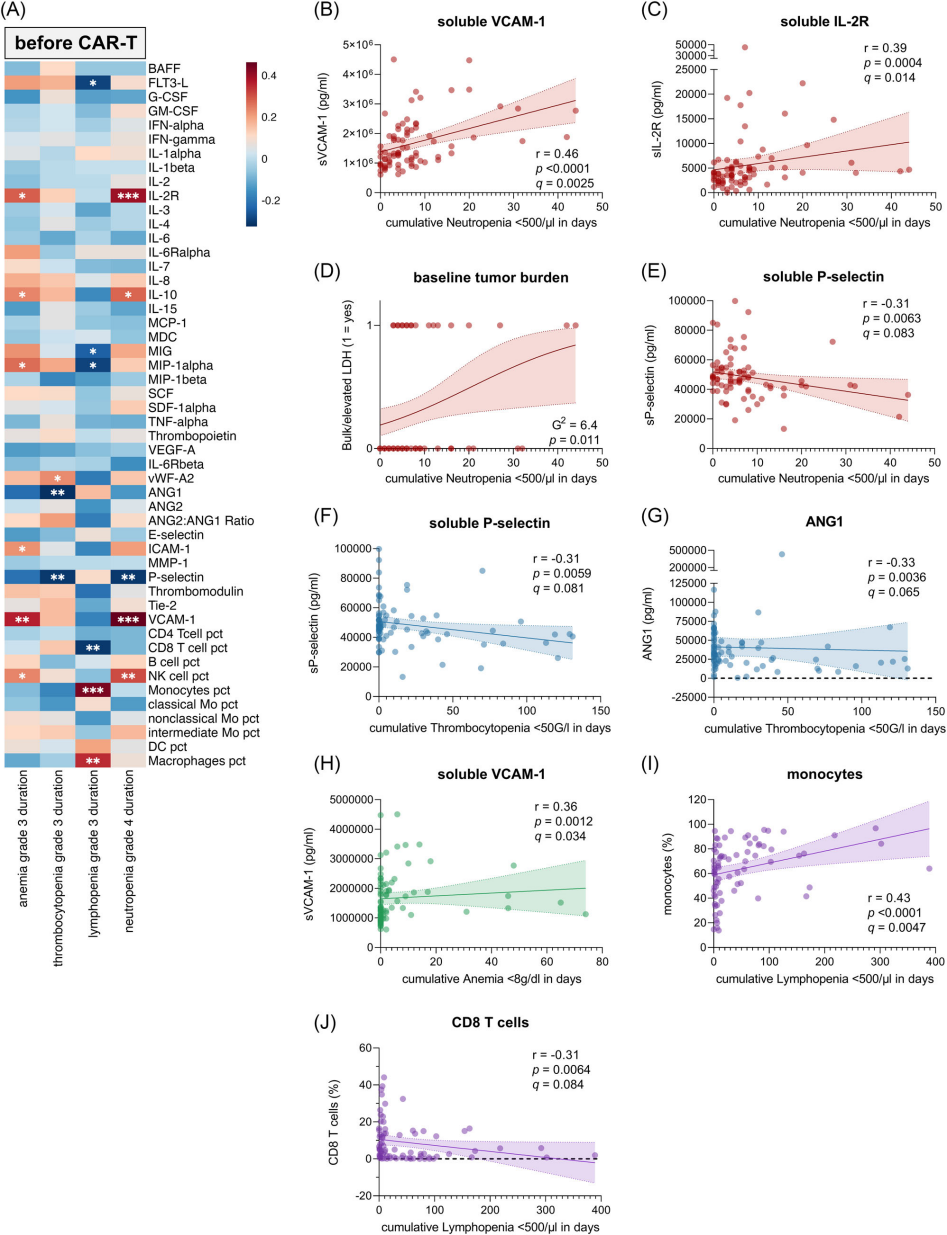
**Baseline factors associated with severe hematotoxicity after chimeric antigen receptor (CAR)‐T**. **(A)** Spearman correlation analysis of the influence of cytokines and flow cytometry markers at baseline (before start of lymphodepletion; BS2) and cumulative Grade ≥3 anemia, Grade ≥3 thrombocytopenia, Grade ≥3 lymphopenia, and Grade ≥4 neutropenia. The Spearman correlation coefficient (*r* = scalebar) and the respective P values are depicted in the heatmap, with red indicating positive correlation and blue indicating negative correlation (*P < 0.05, **P < 0.01, and ***P < 0.001). Univariate analysis of the influence of baseline soluble VCAM‐1 **(B)**, soluble IL‐2 receptor **(C)**, baseline tumor burden **(D)**, and soluble P‐selectin **(E)** on prolonged neutropenia (red). Univariate analysis of the influence of baseline soluble P‐selectin **(F)** and ANG1 **(G)** on prolonged thrombocytopenia (blue) and of soluble VCAM‐1 **(H)** on prolonged anemia (green). Univariate analysis of the influence of baseline monocytes (as percentage of HLA‐DR^+^ CD45^+^ leukocytes) **(I)** and CD8 T cells (as percentage of CD45^+^ leukocytes) **(J)** on prolonged lymphopenia (purple). The influence of baseline tumor burden was analyzed by simple logistic regression and quantified by the likelihood ratio test (G2). For the other variables, best‐fit line (bold line) and 95% confidence intervals (dashed line) were calculated by simple linear regression. The Spearman correlation coefficient (*r*) and respective P‐ and *q*‐values (after adjustment for multiple testing) are depicted for each tested marker. Shown are selected markers that fulfilled a *q*‐value cutoff of <0.1. DC, dendritic cells; Mo, monocytes; pct, percentage.

Next, we investigated baseline factors (before lymphodepletion, BS2) associated with CAR‐T‐associated hematotoxicity. Several markers demonstrated significant correlations (Figure 3A), with 11 markers remaining significant after adjustment for multiple testing (Figures 3B, C, and E–J; S2G,H). Notably, endothelial markers at baseline also showed a strong link with prolonged cytopenias after CAR‐T. For instance, high baseline sVCAM‐1 showed the strongest overall correlation with prolonged neutropenia (*r* = 0.46, P < 0.0001) and anemia (*r* = 0.36, P = 0.0012) (Figure 3B,H). Additionally, low baseline soluble P‐selectin was linked with prolonged neutropenia and thrombocytopenia and low baseline ANG1 with prolonged thrombocytopenia after CAR‐T (Figure 3E–G).

Furthermore, elevated baseline sIL‐2R showed a significant correlation with prolonged neutropenia (*r* = 0.39, P = 0.0004) (Figure 3C). Among inflammatory markers currently incorporated in clinical models, elevated baseline ferritin (*r* = 0.39, P = 0.0005) and CRP (*r* = 0.36, P = 0.011) showed significant associations, underscoring the prognostic relevance of a proinflammatory state before CAR‐T (Figure S4A,B). In contrast, apart from baseline hemoglobin (*r* = –0.49, P < 0.0001) and lactate dehydrogenase (LDH) (*r* = 0.28, P = 0.015), other markers currently used in clinical models, including baseline platelet count, absolute neutrophil count (ANC) and creatinine did not demonstrate a significant correlation with prolonged neutropenia in our cohort (Figure S4C–E, G, and H).

We also analyzed the influence of patient characteristics on prolonged cytopenias, and only found a high tumor burden, reflected by the presence of bulk or elevated LDH at baseline, to be significantly associated with prolonged neutropenia after CAR‐T (*G* ^2^ = 6.4, P = 0.011) (Figure 3D). Interestingly, a high tumor burden also seemed to be associated with high sIL‐2R levels at baseline (Figure S10G). Strikingly, neither the patients' age, disease entity, sex, high‐risk profiles, number of prior therapy lines or a prior autologous/allogeneic stem cell transplantation (SCT) was linked with prolonged neutropenia (Figure S5A–H). Besides an association between prolonged thrombocytopenia and MM (*G* ^2^ = 6.4, P = 0.011), no other relevant association was found between the mentioned baseline characteristics, especially prior treatments, and prolonged anemia, thrombocytopenia, or lymphopenia.

Since endothelial dysfunction has been reported in the context of severe CRS and ICANS,^18^, ^19^ we analyzed their influence on severe hematotoxicity. Surprisingly, we could not see any association between CRS/ICANS severity or duration nor their treatment with dexamethasone/tocilizumab and prolonged neutropenia, thrombocytopenia, lymphopenia, or anemia after CAR‐T (Figure S5I–L). However, like hematotoxicity, CRS and ICANS were associated with elevated baseline proinflammatory markers, including IL‐1β, IL‐1α, TNF‐α, and IFN‐γ, further supporting the role of a proinflammatory state as a shared pathophysiological feature across CAR‐T‐related toxicities (Figure S3B).

Finally, a high percentage of monocytes and macrophages and a low percentage of CD8 T cells (concomitantly high CD4:CD8 ratio, *r* = 0.3, P = 0.0087) at baseline were found to be associated with prolonged lymphopenia after CAR‐T (Figures 3I–J; S2G). Although monocytes and macrophages are key orchestrators of the cytokine storm,^20^, ^21^ no association was observed between their peripheral blood percentage and the occurrence of CRS, ICANS, or ICAHT.

All in all, endothelial dysfunction, a proinflammatory state, and high tumor burden are key determinants of CAR‐T‐associated hematotoxicity. Elevated baseline sVCAM‐1 and sIL‐2R reflecting these states represent potential biomarkers for identifying patients at high risk of prolonged neutropenia. Notably, these markers exhibited a consistent association with prolonged neutropenia across all CAR‐T products (Figure S6).

### Patients with high baseline levels of either sIL‐2R or sVCAM‐1 have reduced OS after CAR‐T therapy

**Figure 4:**
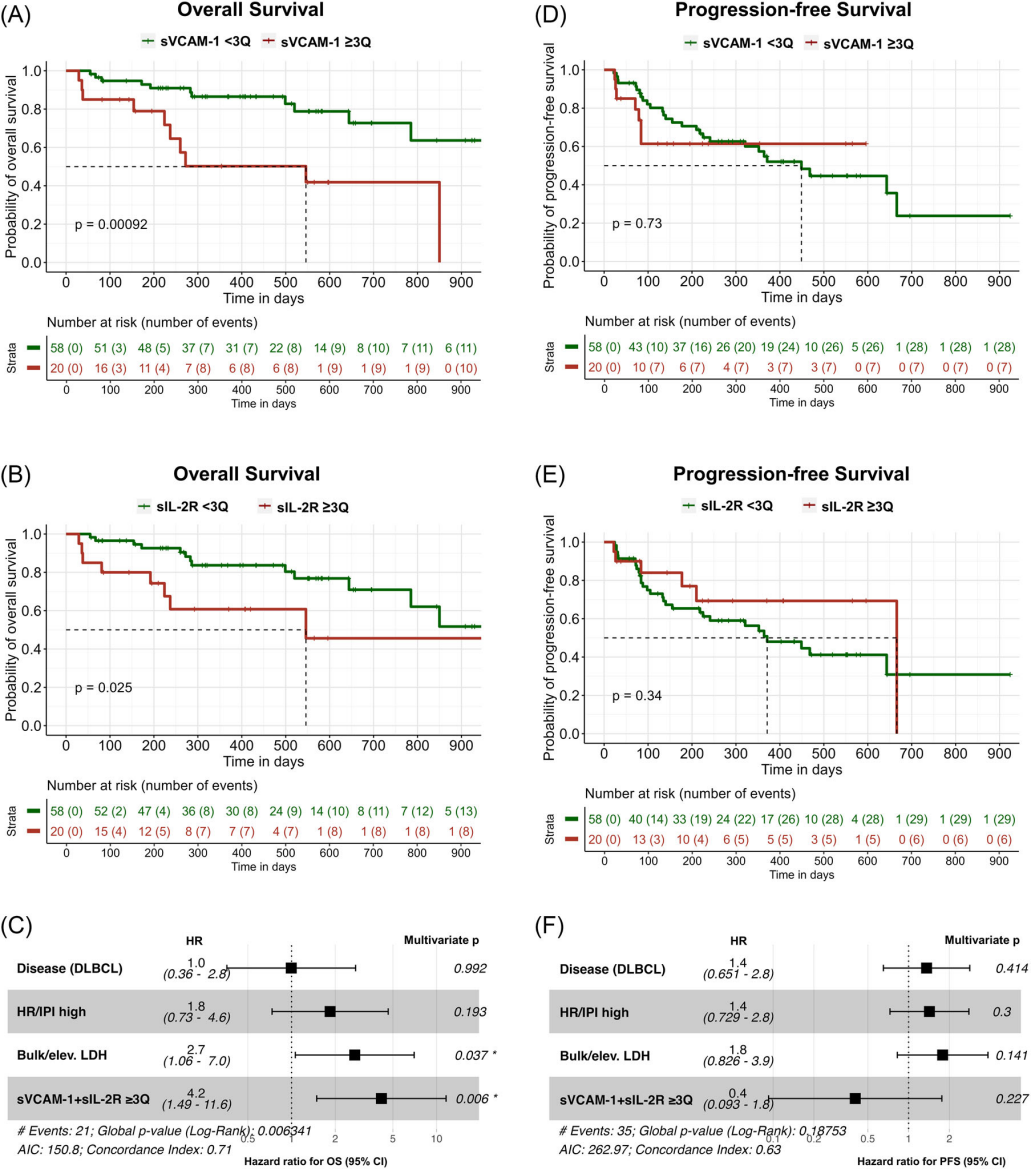
**High baseline sVCAM‐1 and sIL‐2R before chimeric antigen receptor (CAR)‐T is associated with inferior outcome**. Kaplan–Meier curves for overall survival (OS) **(A, B)** and progression‐free survival (PFS) **(D, E)** for patients with high (red) and low (green) sVCAM‐1 **(A, D)** and sIL‐2R **(B, E)** at baseline (before start of lymphodepletion; BS2). Patients with values ≥ 3 quartile of all patients were characterized as high (sIL‐2R ≥ 5398.8 pg/mL; sVCAM‐1 ≥ 2094.0 ng/mL). Dashed lines indicate the median of OS and PFS, respectively. Statistical significance was determined by log‐rank test. **(C, F)** Forest plots showing the results of multivariate Cox regression analysis on the influence of different covariates on OS **(C)** and PFS **(F)**. HR/IPI high, high‐risk cytogenetics (multiple myeloma [MM]) or International Prognostic Index (IPI) ≥ 3 (diffuse large B‐cell lymphoma [DLBCL]); bulk/elev. LDH, presence of bulk or elevated lactate dehydrogenase (LDH) at baseline; sVCAM‐1 + sIL‐2R ≥ 3Q, sVCAM‐1 and sIL‐2R levels above the third quartile; AIC, Akaike information criterion.

Since baseline sVCAM‐1 and sIL‐2R showed the strongest correlation with CAR‐T‐associated hematotoxicity, we further investigated their impact on patient outcome. We therefore separated our cohort in two groups of either high (≥3Q) or low (<3Q) baseline values, with a threshold based on the third quartile of each marker (sIL‐2R ≥ 5398.8 pg/mL; sVCAM‐1 ≥ 2094 ng/mL). Strikingly, patients with high baseline levels of either sVCAM‐1 or sIL‐2R had significantly reduced OS after CAR‐T therapy (sVCAM‐1 P = 0.0009; sIL‐2R P = 0.025) (Figure 4A,B). In multivariate Cox regression analysis, elevation of both markers (sVCAM‐1 and sIL‐2R ≥ 3Q) had a greater impact on OS than the presence of bulk or elevated LDH, while disease entity and high‐risk profiles, such as high‐risk cytogenetics or high International Prognostic Index (IPI), had no significant effect (Figure 4C). Notably, a similar trend in distinguishing patients with poorer OS was observed when CAR‐T products were analyzed separately (Figure S7A–F). Compared to the impaired OS, neither of the markers seems to affect PFS after CAR‐T therapy (Figure 4D–F). However, ide‐cel patients with high baseline sVCAM‐1 or sIL‐2R showed a trend towards worse PFS (P = 0.15; P = 0.17) (Figure S7G,J).

### Patients with high baseline levels of either sIL‐2R or sVCAM‐1 have prolonged neutropenia, show a more aplastic neutrophil recovery, and develop more severe infections

**Figure 5:**
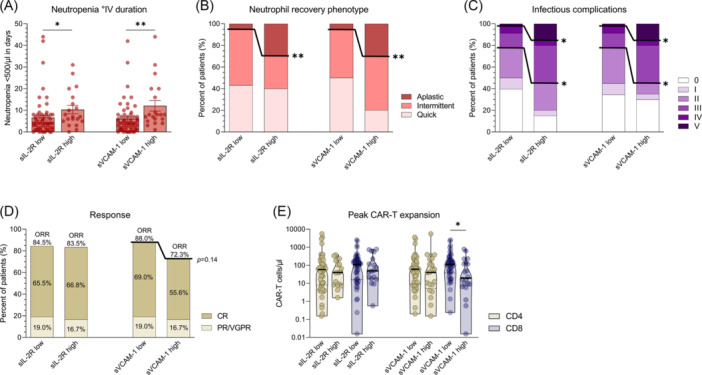
**High sIL‐2R and sVCAM‐1 before chimeric antigen receptor (CAR)‐T is associated with more prolonged neutropenia, a more aplastic neutrophil recovery and more severe infections**. **(A)** Mean cumulative duration of Grade 4 neutropenia between patients with high and low sIL‐2R (10.3 vs. 6.6 days) or sVCAM‐1 (12.1 vs. 6.0 days) at baseline. Whiskers indicate standard error of the mean (SEM). Statistical significance was determined by Mann–Whitney *U* test. **(B)** Percentage of quick, intermittent, and aplastic neutrophil recovery phenotypes between patients with high and low sIL‐2R or sVCAM‐1 at baseline. **(C)** Distribution of infection grades (CTCAE grade) between patients with high and low sIL‐2R or sVCAM‐1 at baseline. **(D)** Overall response rates of patients with high and low sIL‐2R or sVCAM‐1 at baseline subdivided by percentage of patients achieving partial remission (PR)/very good partial remission (VGPR) and complete remission (CR). Statistical significance was determined by Fisher's exact test **(B–D)**. **(E)** Peak CD4 and CD8 CAR‐T expansion (d0 until d14) per µL peripheral blood between patients with high and low sIL‐2R or sVCAM‐1 at baseline as determined by flow cytometry. Statistical significance was determined by Mann–Whitney *U* test. *P < 0.05, **P < 0.01, and ***P < 0.001. Patients with values ≥ 3 quartile of all patients were characterized as high (sIL‐2R ≥ 5398.8 pg/mL; sVCAM‐1 ≥ 2094.0 ng/mL).

We next sought to investigate the reasons for poorer outcomes of patients with high baseline sIL‐2R and sVCAM‐1. Comparing the two groups we could see that patients with high levels of either sIL‐2R or sVCAM‐1 had significantly longer duration of neutropenia (mean duration of Grade 4 neutropenia 6.6 vs. 10.3 days; P = 0.019 for sIL‐2R and 6.0 vs. 12.1 days; P = 0.0016 for sVCAM‐1), a higher percentage of aplastic neutrophil recoveries (5% vs. 30%; P = 0.007 for both) and a higher percentage of severe (Grade ≥3; 22.4% vs. 55%; P = 0.011 for both) and fatal (Grade 5) infections after CAR‐T (Figure 5A–C). Compared to that, patients with high baseline sIL‐2R and sVCAM‐1 did not show significant differences in response rates, although patients with high sVCAM‐1 showed reduced overall and CR rates (both n.s.) and had significantly lower peak expansion of CD8 CAR‐T cells (Figure 5D,E). No significant differences in the occurrence of CRS or ICANS could be found between patients with high and low baseline sIL‐2R and sVCAM‐1 (Figure S8A,B). In addition to analyzing sVCAM‐1 and sIL‐2R individually, we also explored their combined impact by stratifying patients into groups with both markers elevated (≥third quartile) or either marker elevated. These combinations revealed the same trends to those observed for the individual markers, with the high/high group consistently exhibiting the most adverse outcomes in terms of neutrophil recovery phenotype and infection rates (data not shown). Of note, when looking at MM patients only, patients with high baseline sIL‐2R showed a trend towards reduced minimal residual disease negative CR rates (P = 0.052), and patients with high baseline sVCAM‐1 trend to have lower overall response rates (P = 0.088) (Figure S8C).

Taken together, elevated baseline sIL‐2R and sVCAM‐1 identify patients with inferior outcomes after CAR‐T therapy characterized by prolonged neutropenia, aplastic neutrophil recovery, and severe infections.

### sIL‐2R and sVCAM‐1 at baseline have predictive value for adverse outcomes of CAR‐T and can further improve existing models

**Figure 6:**
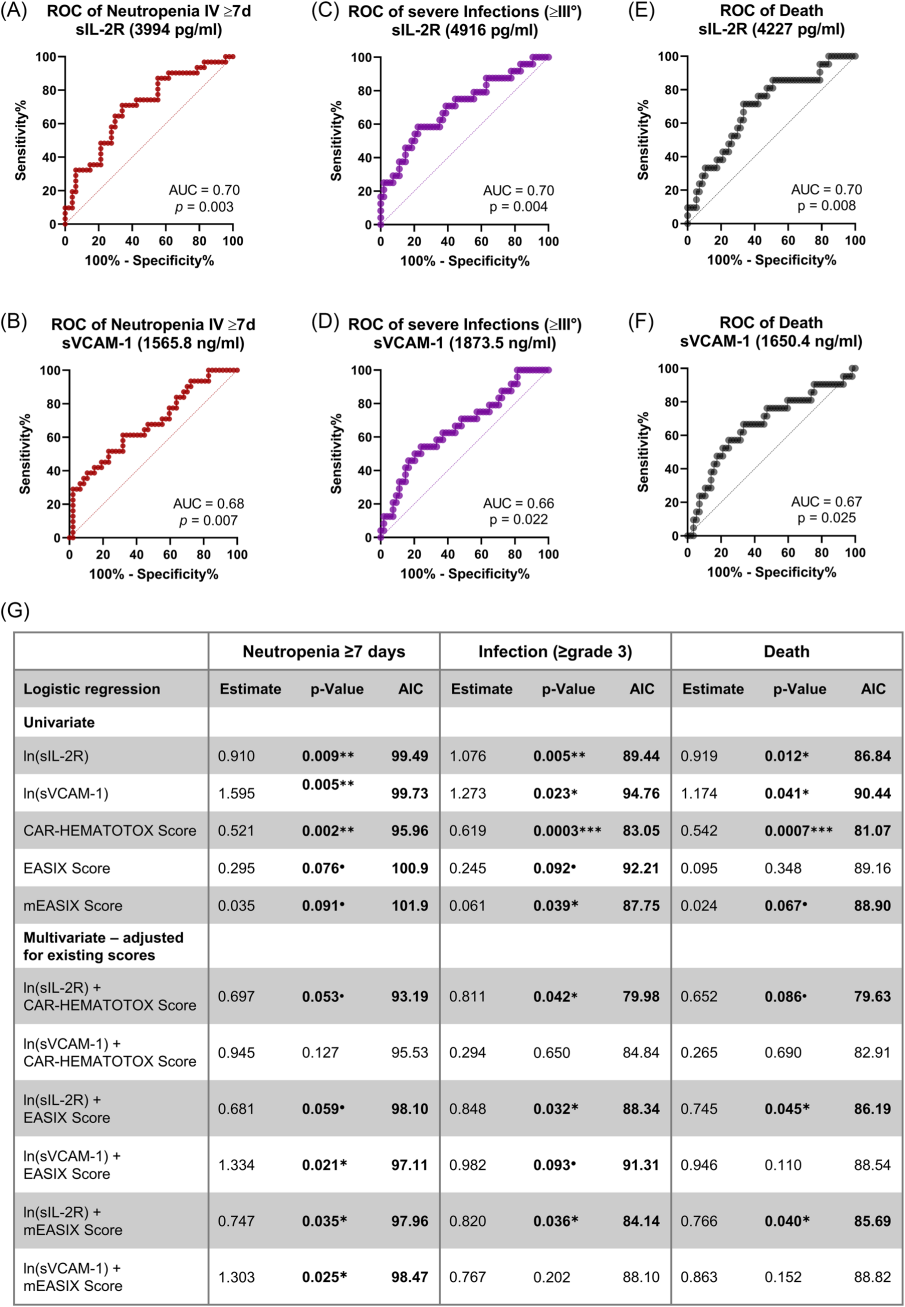
**Predictive value of baseline sIL‐2R and sVCAM‐1 on adverse outcomes after chimeric antigen receptor (CAR)‐T**. Receiver operating characteristic (ROC) curves of the influence of baseline sIL‐2R **(A)** and sVCAM‐1 **(B)** on the occurrence of prolonged Grade 4 neutropenia of ≥7 days (31 events). ROC curves of the influence of baseline sIL‐2R **(C)** and sVCAM‐1 **(D)** on the occurrence of severe infections (≥Grade 3) (24 events). ROC curves of the influence of baseline sIL‐2R **(E)** and sVCAM‐1 **(F)** on the occurrence of death (21 events). For ROC curves, the respective area under the curve (AUC/AUROC) and P‐value are depicted. The concentration in brackets depicts the cutoff optimizing the Youden index. **(G)** Tabular summary of logistic regression analyses, including univariate models for baseline sIL‐2R, sVCAM‐1, CAR‐HEMATOTOX‐, EASIX‐, and mEASIX‐score, as well as multivariate models combining either sIL‐2R or sVCAM‐1 with CAR‐HEMATOTOX‐, EASIX‐, or mEASIX‐score. For each model, the estimate, P‐value, and Akaike information criterion (AIC) are reported. •P < 0.1, *P < 0.05, **P < 0.01, ***P < 0.001.

The primary aim of this study was to identify novel markers for adverse outcomes of CAR‐T therapy and thus improve risk stratification. We therefore tested the predictive capacity of baseline sIL‐2R and sVCAM‐1 on adverse outcomes of CAR‐T therapy in ROC analysis. Both markers showed significant predictive value for prolonged Grade 4 neutropenia (≥7 days), severe infections (Grade ≥3), and death after CAR‐T (Figure 6A–F). Importantly, the integration of these biomarkers into existing risk models, including EASIX, mEASIX, and CAR‐HEMATOTOX, consistently improved their predictive performance, as reflected by increased AUCs and significance levels across nearly all endpoints (Table S5). Notably, this improvement was seen across all CAR‐T products (Figure S9), underscoring the broad applicability of these biomarkers.

To further assess their independent prognostic value, we performed univariate and multivariate logistic regression analyses. In univariate models, both sIL‐2R and sVCAM‐1 were significantly associated with all three endpoints: prolonged neutropenia (P < 0.01 for both), severe infections (P = 0.005 and P = 0.023), and death (P < 0.05 for both) (Figure 6G). The CAR‐HEMATOTOX score showed strong associations across all endpoints, while EASIX and mEASIX demonstrated weaker or borderline associations.

In multivariate models adjusted for the CAR‐HEMATOTOX, sIL‐2R retained independent prognostic value for severe infections (P = 0.042) and showed trends toward significance for neutropenia (P = 0.053) and death (P = 0.086) (Figure 6G). sVCAM‐1, while not reaching statistical significance in multivariate models adjusted for the CAR‐HEAMTOTOX, did not worsen model performance, as reflected by stable AIC values across all endpoints. When adjusting for EASIX or mEASIX, both biomarkers demonstrated improved prognostic value: sIL‐2R significantly strengthened associations with all endpoints, while sVCAM‐1 particularly reinforced the association with prolonged neutropenia. Taken together, these findings suggest that both biomarkers provide incremental prognostic information beyond existing risk scores.

We further examined the prognostic value of our markers compared to individual score components. In multivariate models adjusting for ferritin or hemoglobin, the only components with strong and consistent univariate associations, sIL‐2R remained significantly associated with infections and showed trends for neutropenia and death, while sVCAM‐1 retained significance for neutropenia and showed borderline associations with infections (Table S4). This supports the additional prognostic value of our biomarkers beyond established score components.

To account for potential confounding, we adjusted for prior autologous or allogeneic SCT and the presence of bulk or elevated LDH. sIL‐2R remained significantly associated with all three endpoints, and sVCAM‐1 retained significance for neutropenia and infections, with a trend for death (Table S4). Finally, we also included the underlying disease and CAR‐T product in multivariate models. Both markers remained significantly associated with almost all endpoints after adjustment, confirming their robust and independent prognostic value across different disease entities and CAR‐T products.

Together, these findings support sIL‐2R and sVCAM‐1 as robust, independent prognostic markers that can enhance current risk models and inform clinical decision‐making.

## DISCUSSION

Our study identifies endothelial dysfunction and a proinflammatory state as key determinants of severe hematotoxicity and adverse outcomes after CAR‐T therapy. We further identified baseline sVCAM‐1 and sL‐2R as valuable molecular markers reflecting these states and proved their applicability to predict such adverse outcomes.

So far, several biomarkers and models have been proposed for the prediction of adverse outcomes after CAR‐T therapy.^3^, ^19^, ^22^, ^23^, ^24^, ^25^, ^26^, ^27^ The EASIX‐score, originally developed in the context of allogeneic SCT, focuses on surrogate blood markers to reflect endothelial activation.^23^, ^25^, ^26^, ^28^ However, apart from platelet count, its markers have no direct mechanistic link to endothelial dysfunction. Other models such as the CAR‐HEMATOTOX are based on routine lab markers to reflect hematopoietic reserve and a baseline proinflammatory state as additional key factors contributing to CAR‐T‐associated hematotoxicity in particular.^3^, ^29^, ^30^ At the cost of being easy‐to‐use, most models only comprise baseline routine lab markers and therefore lack mechanistic insights.

Foremost, predictive markers need to be broadly applicable and reproducible. Therefore, we aimed to develop markers based on a heterogeneous cohort of MM and DLBCL patients, the tumor entities currently most commonly treated with CAR‐T cells in adults.^5^ Importantly sVCAM‐1 and sIL‐2R showed comparable trends for all CAR‐T products (Figures S7 and S9). Recent studies have linked elevated baseline EASIX with severe late ICAHT, severe infections, and inferior PFS and OS in patients treated with ide‐cel.^31^ While our findings align with these observations, we observed less pronounced associations, likely reflecting differences in patient composition and inferior model performance for axi‐cel. Among existing models, the CAR‐HEMATOTOX remains the most established predictor of CAR‐T‐associated hematotoxicity, having been originally developed in DLBCL patients with subsequent validation in different tumor entities.^3^, ^15^, ^29^, ^30^ Although some of its features did not show the same correlations in our cohort, the overall score significantly correlated with prolonged neutropenias and proved to be a valuable predictor (Figures S4 and S9). However, given its development in DLBCL patients only, we and others have observed that it tends to underperform in MM patients (Figure S9).^32^ The addition of baseline sVCAM‐1 or sIL‐2R could compensate for the deficiencies of existing scores without significantly compromising their predictive power for other CAR‐T products (Figure S9) and thus improving their applicability for a broader spectrum of patients. The integration of sIL‐2R or sVCAM‐1 into the CAR‐HEMATOTOX, EASIX, and mEASIX improved their discriminatory capacity primarily by increasing specificity for the endpoints prolonged neutropenia, severe infections, and death. This added specificity, likely by reflecting distinct pathophysiological processes, is clinically relevant, as it may help reduce false positives and improve pretreatment risk assessment and clinical decision‐making. Importantly, both markers demonstrated consistent and independent prognostic value across almost all endpoints, even after adjusting for key clinical variables including the underlying disease, CAR‐T product type, prior autologous or allogeneic SCT, and indicators of disease burden such as bulk or elevated LDH (Table S4).

VCAM‐1 is an adhesion molecule upregulated during endothelial activation and facilitates leukocyte adhesion and transendothelial migration via VLA‐4.^33^ Its soluble form (sVCAM‐1) is released through proteolytic cleavage, a process enhanced by IL‐1β and TNF‐α during inflammation.^34^ Increased levels of sVCAM‐1 have been associated with numerous pathological conditions such as immunological disorders, cancer, and cardiovascular disease.^33^, ^35^ Although sVCAM‐1 is mechanistically connected to endothelial dysfunction and cardiovascular disease, no cardiovascular events were observed in our cohort, likely reflecting its low incidence following CAR‐T therapy. sVCAM‐1 was also found to be elevated in advanced stage non‐Hodgkin's lymphoma and MM where it has been associated with poorer survival.^36^, ^37^ In contrast to these findings, in our cohort elevated sVCAM‐1 levels only correlated with increased baseline R‐ISS and presence of extramedullary disease (Figure S10A,B) but did not show an association with Ann‐Arbor stage, IPI, or tumor burden. Besides that, elevated levels of sVCAM‐1 have also been observed in patients with myelodysplastic syndromes, where it is associated with disease severity.^38^ In line with this, a defective vascular niche contributes to the pathogenesis of myelodysplasia.^39^, ^40^ Taken together, sVCAM‐1 has prognostic impact not only by providing insight into advanced disease stages but also by reflecting preexisting endothelial dysfunction and damage to the bone marrow niche and therefore reflects key events of the adverse outcome pathway of hematotoxicity after CAR‐T (Figure S10C–F).

The IL‐2 receptor exists as a low‐affinity dimer (IL‐2Rβ/CD122 and IL‐2Rγ/CD132) and a high‐affinity trimer, which includes IL‐2Rα/CD25. High levels of the trimeric IL‐2R are transiently expressed on activated T cells and constitutively expressed on regulatory T cells.^41^ IL‐2Rα can be shed and released as a soluble form (sIL‐2R), a process that has been described for activated T cells, regulatory T cells, dendritic cells, and monocytes.^41^, ^42^ Elevated levels of sIL‐2R have been observed in autoimmune and inflammatory diseases, infections, solid cancers, and hematological malignancies and are thought to reflect sustained immune activation and a proinflammatory state.^41^ Furthermore, sIL‐2R has been proposed as a biomarker for IEC‐HS, a rare but severe complication of CAR‐T therapy.^43^ In our cohort, although some patients exhibited elevated sIL‐2R alongside hyperferritinemia and hypercytokinemia, features often seen in HLH‐like syndromes,^44^, ^45^ no cases of IEC‐HS were observed (Table 2). This suggests that while these markers reflect a hyperinflammatory state, as a potential mechanistic overlap between ICAHT and IEC‐HS, their presence alone does not necessarily indicate overt hemophagocytic pathology.

In patients with untreated aggressive B‐cell lymphoma, elevated sIL‐2R levels were associated with advanced disease stage and were linked to poorer OS.^46^, ^47^ Compared to that, newly diagnosed MM patients with high sIL‐2R had worse PFS but showed no differences in OS.^48^, ^49^ These reports are in line with our findings that elevated baseline sIL‐2R is associated with high tumor burden, reflected by elevated LDH or bulky disease (Figure S10G). Furthermore, high baseline sIL‐2R was strongly associated with high levels of proinflammatory cytokines (GM‐CSF, TNF‐α, and IL‐1β) at baseline (Figure S10I–K) and early after CAR‐T (TNF‐α *r* = 0.46, P < 0.0001; IL‐1β *r* = 0.43, P < 0.0001; GM‐CSF *r* = 0.39, P = 0.0005). All in all, elevated baseline sIL‐2R not only characterizes patients with high tumor burden and advanced disease stages but also reflects a proinflammatory baseline state and predicts a hyperinflammatory response after CAR‐T (Figure S10L).

The results of our study are limited by its retrospective design and the use of preselected biomarkers based on existing literature, rather than an unbiased screening approach, which may have constrained the discovery of novel associations. The small sample size, though reflective of the limited number of CAR‐T patients available, raises concerns about the generalizability and the potential for overfitting. Additionally, validation in larger, independent cohorts is necessary to confirm their predictive value and clinical applicability, which is currently underway.

In summary, our study identified endothelial dysfunction and a proinflammatory state as key risk factors of severe hematotoxicity and adverse patient outcomes after CAR‐T therapy. sVCAM‐1 and sIL‐2R are suitable molecular markers to detect these unfavorable conditions and reflect distinct key events within the irAOP of hematotoxicity. These markers could therefore help to improve risk assessment and to guide early or prophylactic interventions for patients undergoing CAR‐T therapy.

## AUTHOR CONTRIBUTIONS

**Lukas Scheller**: Conceptualization; data curation; investigation; formal analysis; methodology; visualization; writing—original draft; writing—review and editing. **Xiang Zhou**: Resources; data curation; investigation; formal analysis; writing—review and editing. **Henry Loeffler‐Wirth**: Formal analysis; methodology; writing—review and editing. **Markus Kreuz**: Formal analysis; methodology; writing—review and editing. **Sofie‐Katrin Kadel**: Resources. **Sven Schimanski**: Resources. **Hannah Schulze**: Resources. **Anna Ruckdeschel**: Resources. **Florian Eisele**: Resources. **Verena Konetzki**: Methodology; investigation; formal analysis. **Maria Jornet Culubret**: Methodology; investigation; formal analysis. **Maximilian Merz**: Validation. **Julia Mersi**: Resources. **Johannes Waldschmidt**: Resources. **Sophia Danhof**: Resources; methodology; visualization; writing—review and editing. **Ulrike Köhl**: Funding acquisition; project administration. **K. Martin Kortüm**: Resources; investigation; writing—review and editing. **Leo Rasche**: Investigation; resources; writing—review and editing. **Hermann Einsele**: Resources; investigation; writing—review and editing; supervision. **Johannes Düll**: Resources; investigation; project administration. **Max S. Topp**: Resources; project administration; investigation. **Michael Hudecek**: Supervision; resources; funding acquisition; writing—review and editing. **Kristin Reiche**: Conceptualization; supervision; project administration; writing—review and editing. **Miriam Alb**: Conceptualization; supervision; project administration; investigation; data curation; methodology; writing—original draft; writing—review and editing.

## CONFLICT OF INTEREST STATEMENT

Lukas Scheller, Sophia Danhof, and Miriam Alb are listed as inventors on patent applications filed by the University of Würzburg, Würzburg, Germany. Michael Hudecek is listed as an inventor on patent applications and granted patents related to CAR‐T technologies that have been filed by the Fred Hutchinson Cancer Research Center, Seattle, Washington, and by the University of Würzburg, Würzburg, Germany. Michael Hudecek is a co‐founder and equity owner of T‐CURX GmbH, Würzburg, Germany. Michael Hudecek received honoraria from Celgene/BMS, Janssen, and Kite/Gilead. Henry Loeffler‐Wirth received speaker's honoraria from ThermoFisher Scientific. Sophia Danhof received honoraria from Celgene/BMS and Sanofi. K. Martin Kortüm declares research funding and honoraria from Janssen and is supported by the DFG and the Stifterverband. Ulrike Köhl received consultant and/or speaker fees from AstraZeneca, Affimed, Glycostem, GammaDelta, Zelluna, Miltenyi Biotec, Novartis Pharma, and BMS. Hermann Einsele and Leo Rasche received honoraria from Pfizer, Amgen, Janssen, Sanofi, and BMS. Johannes Düll and Max S. Topp received honoraria/funding from BMS, Janssen, Gilead‐Kite, and Novartis. Leo Rasche received funding from SkylineDx. The remaining authors declare that the research was conducted in the absence of any commercial or financial relationships that could be construed as a potential conflict of interest.

## ETHICS STATEMENT

This study was conducted with approval from the institutional review board (ethics committee) and informed consent from the patients.

## FUNDING

L.S. received funding from the Interdisciplinary Center for Clinical Research (IZKF), grant number Z‐2_CSP_2022‐27. The authors are supported by the European Union's Innovative Medicines Initiative 2 Joint Undertaking under grant agreement no. 853988 (imSAVAR to L.S., H.L.‐W., M.K., U.K., M.H., K.R., and M.A.) and no. 945393 (T2EVOLVE to M.K., M.H., H.E., and K.R.). This Joint Undertaking receives support from the European Union's Horizon 2020 research and innovation program and EFPIA and JDRF INTERNATIONAL. The funders had no role in study design, data collection, and analysis. Neither Innovative Medicines Initiative 2 nor the European Union, EFPIA, or JDRF INTERNATIONAL is responsible for any use that may be made of the information contained therein. K.R., M.H., M.K., and U.K. were supported by SaxoCell (BMBF Clusters4Future). The authors have been supported by the patient advocacy group “Hilfe im Kampf gegen den Krebs e.V.”, Würzburg, Germany, and “Forschung hilft” ‐ Stiftung zur Förderung der Krebsforschung an der Universität Würzburg (L.S., H.E., M.H., M.A., and S.D.). Further, the authors have been supported by the German Research Foundation (Deutsche Forschungsgemeinschaft, DFG, TRR 221, subproject A03 to M.H., H.E., and S.D.; and TRR338, subproject A02 to M.H. and B05 to S.D.), the Bavarian Center for Cancer Research (Bayerisches Zentrum für Krebsforschung, Leuchtturm Zelluläre Immuntherapie to M.H. and H.E.), and the National Center for Tumor Diseases (NCT WERA to L.S., S.D., H.E., M.H., and M.A.). M.H. acknowledges funding by the German Ministry for Science and Education (BMBF, Bundesministerium für Bildung und Forschung, grant #13N15986 Imagine and grant #01EN2306A ROR2). The authors received support from the Paula and Rodger Riney Foundation. Open Access funding enabled and organized by Projekt DEAL.

## Supporting information

Supporting Information.

## Data Availability

The data that support the findings of this study are available from the corresponding author upon reasonable request.
