# Supplementary material for: Endothelial dysfunction and proinflammatory state determine severe hematotoxicity and inferior outcome of CAR‐T therapy
Source: Hemasphere. 2025 Dec 8;9(12):e70267. doi: 10.1002/hem3.70267 (PMC12684805; doi:10.1002/hem3.70267)
Supplement: Supplementary file 1 — Supporting Information. [file HEM3-9-e70267-s001.pdf]

Supplemental data

Study cohort description

This single-center study included patients treated with approved CAR-T products between November 2021 and August 2024 at Universitätsklinikum Würzburg. Fifty-four patients had relapsed/refractory multiple myeloma (MM) treated with ide-cel (n=31) or cilta-cel (n=23) and 24 patients had relapsed/refractory B-cell lymphoma (DLBCL) treated with axi-cel. Forty-eight patients (61%) were male and the median age was 64 years (range 22-79 years). The MM patients included 21 (39%) patients with high-risk cytogenetics and 16 (30%) with extramedullary disease. MM patients received a median of five prior lines of therapy (range 2-10), including autologous and allogeneic stem cell transplantation (SCT) in 53 (98%) and 7 (13%) of the patients, respectively. None of the patients with previous allogeneic stem cell transplantation had ongoing GvHD. Thirty-nine MM patients (72%) were penta-exposed. Within the DLBCL patients, 10 patients (42%) had an international prognostic index (IPI) ≥3. The DLBCL patients received a median of two prior therapy lines (range 1-5), including five patients (21%) with prior autologous SCT. Of the five patients with prior autologous SCT, three were treated before axi-cel received approval for second-line use, and two experienced late relapse (>12 months) after first-line therapy. Twelve DLBCL patients (50%) had primary refractory disease and 9 (38%) had early relapse after frontline treatment. Fourteen DLBCL patients (58%) showed a high baseline tumor burden before CAR-T therapy characterized by either elevated LDH levels or presence of bulky disease, compared to 9 (17%) MM patients with elevated LDH levels at baseline. Prior to the initiation of lymphodepleting chemotherapy, seven patients (9%) had pre-existing grade ≥3 neutropenia, including one patient (<1%) with grade 4 neutropenia. Additionally, grade ≥3 thrombocytopenia, anemia, and lymphopenia were present in five (6%), three (4%), and 18 (23%) patients, respectively. The use of bridging therapies, incidence of adverse events and incidence and causes of deaths are summarized in supplemental **Table S1-S3**.

Supplemental Methods

Sampling and cell processing

Peripheral blood mononuclear cells (PBMC) were isolated from EDTA-blood by density gradient centrifugation using Pancoll human separating solution (density: 1.077 g/ml; PAN-Biotech GmbH, Aidenbach, Germany). PBMC were counted using a Neubauer counting chamber and trypan blue for dead cell exclusion. PBMC were cryopreserved for subsequent downstream analyses using a freezing protocol by 10X Genomics (Pleasanton, CA, USA).

Flow cytometry

Surface markers were stained directly in whole blood samples for 30 minutes (min) at room temperature (RT). After one washing step with MACSQuant® Running Buffer (Miltenyi Biotec, Bergisch Gladbach, Germany), red blood cells were lysed by incubation in 1X BD Pharm Lyse™ Lysing Buffer (BD Biosciences, Heidelberg, Germany) for 15 min at RT as recommended by the manufacturer. After an additional washing step, live/dead cell staining was carried out using the 7-AAD staining solution from Miltenyi Biotec. For CAR-T cell detection, whole blood was incubated with 1X BD Pharm Lyse™ Lysing Buffer for 15 min at RT to lyse red blood cells and washed once with MACSQuant® Running Buffer. Then, samples were incubated with the respective CAR detection reagent for 10 minutes at RT. After that, samples were washed and stained with antibodies for the following surface markers: CD45, CD3, CD4 and CD8 for 10 minutes at RT. After a final washing step, live/dead cell staining was carried out with the 7-AAD staining solution from Miltenyi Biotec. Cells with the forward and side light scatter properties of leukocytes were acquired on a MACSQuant10 analyzer. Immune cell subsets were analyzed as frequencies and absolute cell counts of $CD45^+$, live leukocytes.

Supplemental Table 1: Bridging therapies

Bridging therapies according to Frenking et al.[13]	ide-cel n=31	axi-cel n=24	cilta-cel n=23
**Extensive cytotoxic**[†]	10 (32%)	3 (13%)	6 (26%)
**Intermediate cytotoxic**[‡]	7 (23%)	1 (4%)	6 (26%)
**Non-cytotoxic**[§]	9 (29%)	8 (33%)	4 (17%)
**Irradiation**	0	4 (17%)	1 (4%)
**No Bridging**	5 (16%)	8 (33%)	5 (22%)
**Not available**	0	0	1 (4%)

[†] ≥3 cytotoxic agents e.g. PACE based regimens or high dose chemotherapy followed by autologous HCT for MM or CHOP for DLBCL
[‡] 1-2 cytotoxic agents e.g. KCD for MM or R-Bendamustin, R-GDP for DLBCL
[§] no cytotoxic agents e.g. KRD for MM, R-Polatuzumab for DLBCL

Supplemental Table 2: Incidence of adverse events after CAR-T per product

Adverse events	ide-cel n=31	axi-cel n=24	cilta-cel n=23
**CRS any grade – no. (%)**[†]	31 (100%)	23 (96%)	17 (74%)
0°	0	1 (4%)	6 (26%)
I°	15 (48%)	6 (25%)	12 (52%)
II°	16 (52%)	16 (67%)	5 (22%)
III°	0	1 (4%)	0
IV°	0	0	0
V°	0	0	0
**ICANS any grade – no. (%)**[†]	3 (10%)	12 (50%)	1 (4%)
0°	28 (90%)	12 (50%)	22 (96%)
I°	3 (10%)	4 (17%)	0
II°	0	1 (4%)	1 (4%)
III°	0	6 (25%)	0
IV°	0	1 (4%)	0
V°	0	0	0
Cranial nerve palsy	0	0	2 (9%)
MNT	0	0	1 (4%)
**Infections any grade – no. (%)**[‡]	23 (74%)	12 (50%)	17 (74%)
0°	8 (26%)	12 (50%)	6 (26%)
I°	3 (10%)	1 (4%)	3 (13%)
II°	11 (35%)	5 (21%)	5 (22%)
III°	4 (13%)	3 (13%)	8 (35%)
IV°	3 (10%)	1 (4%)	1 (4%)
V°	2 (6%)	2 (8%)	0
**Cause of infection**			
Viral	14 (61%)	6 (50%)	9 (53%)
Bacterial	9 (39%)	5 (42%)	6 (35%)
Fungal	1 (4%)	2 (17%)	0
Unknown	2 (9%)	3 (25%)	3 (18%)

**Cytopenia any grade – no. (%)‡**	31 (100%)	23 (96%)	23 (100%)
Anemia CTC ≥3	16 (52%)	13 (54%)	7 (30%)
Thrombocytopenia CTC ≥3	21 (68%)	12 (50%)	13 (57%)
≥3 for ≥14 days	13 (42%)	4 (17%)	9 (39%)
Mean cumulative days of thrombocytopenia <50 G/l (range)	23.7 (0-131)	7.5 (0-71)	30.0 (0-129)
Transfusion required (pRBC or platelets)	14 (45%)	14 (58%)	10 (44%)
Lymphopenia CTC ≥3	29 (94%)	23 (96%)	21 (91%)
≥3 for ≥14 days	17 (55%)	13 (54%)	8 (35%)
Mean cumulative days of lymphopenia <500 /µl (range)	45.9 (0-389)	54.4 (1-302)	52.6 (0-292)
Neutropenia CTC ≥3	31 (100%)	23 (96%)	23 (100%)
≥3 for ≥14 days	15 (48%)	8 (33%)	8 (35%)
Mean cumulative days of neutropenia <1000 /µl (range)	21.3 (3-73)	14.7 (0-68)	15.8 (1-78)
Neutropenia CTC ≥4	28 (90%)	22 (96%)	20 (87%)
≥4 for ≥14 days	5 (16%)	2 (9%)	4 (17%)
≥4 for ≥21 days	2 (7%)	1 (4%)	3 (13%)
Mean cumulative days of neutropenia <500 /µl (range)	6.9 (0-42)	7.5 (0-27)	8.6 (0-44)
G-CSF Support d1-14	26 (84%)	22 (92%)	19 (83%)
G-CSF Support >d14	12 (39%)	8 (33%)	10 (44%)
TPO agonists received	3 (10%)	0	2 (9%)
Autologous stem cell boost	0	0	2 (9%)
Allogeneic stem cell transplantation	1 (4%)	0	0
CAR-HEMATOTOX score high[3] (≥2)	8 (26%)	9 (38%)	9 (39%)
**Neutrophil Recovery type§**			
quick	10 (32%)	13 (54%)	10 (44%)
Intermittent	18 (58%)	8 (33%)	10 (44%)
aplastic	3 (10%)	3 (13%)	3 (13%)
**Secondary malignancies**			
Myelodysplastic syndrome (MDS)	1 (3%)	1 (4%)	0
Acute myeloid leukemia (AML)	1 (3%)	0 (0%)	1 (4%)

† As per ASTCT consensus criteria.
‡ As per CTCAE version 5.0 criteria.
§ Quick: sustained neutrophil recovery without second dip below ANC <1000/µL; intermittent: neutrophil recovery (ANC >1500 cells per µL) followed by a second dip with ANC <1000/µL after day 21; aplastic: continuous severe neutropenia (ANC <500/µL) for ≥14 days

52 **Supplemental Table 3: Incidence and causes of deaths after CAR-T per product**

**Causes of death**	**ide-cel n=31**	**axi-cel n=24**	**cilta-cel n=23**
Disease progression	3 (10%)	4 (17%)	0
Infection after CAR-T	2 (6%)	2 (8%)	0
Infection after subsequent therapy	3 (10%)	2 (8%)	0
Intracranial hemorrhage	0	0	1 (4%)
Secondary MDS/AML	0	1 (4%)	1 (4%)
Unknown	1 (3%)	0	1 (4%)

Supplemental Table 4: Univariate and multivariate logistic regression analysis

	Neutropenia ≥7 days			Infection (≥grade 3)			Death		
	Estimate	p-value	AIC	Estimate	p-value	AIC	Estimate	p-value	AIC
**Univariate**									
CRP	0.090	0.148	105.8	0.319	0.033*	88.3	0.073	0.141	92.5
ln(Ferritin)	0.665	0.001**	95.5	0.443	0.019*	94.3	0.800	0.0006***	79.6
Hemoglobin	-0.447	0.003**	98.3	-0.317	0.031*	95.2	-0.606	0.001**	80.7
Platelets	-0.003	0.227	107.3	-0.005	0.131	97.8	-0.003	0.276	93.6
ANC	-0.048	0.753	108.7	-0.170	0.332	99.3	-0.340	0.098•	91.7
Creatinine	-0.046	0.849	108.8	0.038	0.873	100.3	-0.085	0.777	94.8
LDH	0.002	0.151	106.2	0.003	0.095•	96.7	0.002	0.105	91.9
auto SCT	-0.566	0.280	107.7	0.380	0.518	99.9	-0.526	0.348	94.0
allo SCT	-0.546	0.531	108.4	-0.116	0.895	100.3	0.091	0.918	94.9
Bulk/LDH	1.246	0.016*	102.8	0.539	0.303	99.2	1.417	0.009**	88.0
Disease (MM)	-0.860	0.086•	105.9	0.406	0.463	99.7	-0.742	0.165	93.0
CAR product (cilta-cel)	0.452	0.438	107.2	0.452	0.438	101.1	-1.003	0.172	92.9
CAR product (axi-cel)	1.061	0.063•		-0.205	0.739		0.383	0.508	
**Multivariate**									
ln(sIL-2R) + ln(Ferritin)	0.702	0.055•	93.0	0.915	0.019*	89.3	0.587	0.118	78.8
ln(sVCAM-1) + ln(Ferritin)	1.255	0.038*	92.9	0.986	0.096•	93.4	0.560	0.386	80.8
ln(sIL-2R) + Hemoglobin	0.702	0.047*	94.8	0.934	0.016*	89.3	0.665	0.082•	78.8
ln(sVCAM-1) + Hemoglobin	1.282	0.031*	95.4	1.034	0.080•	94.1	0.585	0.384	82.0
ln(sIL-2R) + auto SCT	0.901	0.010*	100.8	1.116	0.004**	90.4	0.909	0.014*	88.3
ln(sVCAM-1) + auto SCT	1.541	0.007**	101.5	1.464	0.015*	95.2	1.114	0.057•	92.2
ln(sIL-2R) + allo SCT	0.898	0.011*	101.4	1.103	0.005*	91.3	0.959	0.011*	88.5
ln(sVCAM-1) + allo SCT	1.574	0.006**	101.7	1.291	0.024*	96.7	1.214	0.038*	92.3
ln(sIL-2R) + Bulk/LDH	0.844	0.020*	97.6	1.052	0.006**	91.3	0.813	0.032*	84.3
ln(sVCAM-1) + Bulk/LDH	1.470	0.009**	97.5	1.225	0.031*	96.3	1.058	0.079•	86.8
ln(sIL-2R) + Disease	0.877	0.014*	99.7	1.200	0.003**	89.3	0.873	0.019*	88.0
ln(sVCAM-1) + Disease	1.549	0.006**	99.5	1.355	0.019*	95.7	1.123	0.052•	91.0
ln(sIL-2R) + CAR product	0.902	0.017*	101.7	1.258	0.003**	91.1	1.217	0.004**	83.3
ln(sVCAM-1) + CAR product	1.529	0.007**	101.1	1.333	0.021*	97.3	1.261	0.039*	90.3

Supplemental Table 5: ROC analysis on the influence of predictive models on adverse outcomes

ROC Analysis	Neutropenia grade IV ≥7 days		Infection (≥ grade III)		Death	
	AUC	p-value	AUC	p-value	AUC	p-value
CAR-HEMATOTOX	0.67	0.014	0.79	<0.0001	0.74	0.0012
CAR-HEMATOTOX + sIL-2R	0.72	0.0009	0.80	<0.0001	0.77	0.0003
CAR-HEMATOTOX + sVCAM-1	0.69	0.0057	0.78	<0.0001	0.76	0.0005
EASIX	0.69	0.0039	0.67	0.016	0.68	0.017
EASIX + sIL-2R	0.76	<0.0001	0.72	0.002	0.76	0.0004
EASIX + sVCAM-1	0.78	<0.0001	0.73	0.0015	0.78	0.0001
mEASIX	0.72	0.0013	0.68	0.011	0.74	0.001
mEASIX + sIL-2R	0.78	<0.0001	0.73	0.0015	0.78	0.0001
mEASIX + sVCAM-1	0.78	<0.0001	0.73	0.0015	0.78	0.0001

To assess the additive predictive value of our biomarkers, ROC analyses were performed for sIL-2R and sVCAM-1 for each endpoint to determine optimal cutoffs based on the Youden index. For the CAR-HEMATOTOX score, which is a categorized composite model, our biomarkers were added by assigning one additional point if the respective marker exceeded its threshold and were compared to the regular CAR-HEMATOTOX scoring system in ROC analysis. For EASIX and mEASIX, which are continuous scores, we first derived endpoint-specific cutoffs using ROC analysis and then binarized the scores. Composite models were constructed by combining the binarized EASIX/mEASIX scores with our biomarkers, and ROC analysis was used to compare the predictive performance of the binarized original and combined models.

Supplemental Table 6: Flow cytometry antibodies and CAR detection reagents

Marker	Dye	Clone	Manufacturer	Cat. No.
CD56	Pacific Blue	5.1H11	Biolegend	#362520
CD19	BV510	HIB19	Biolegend	#302242
CD16	FITC	3G8	Biolegend	#302006
CD45	PE	2D1	Biolegend	#368510
CD8	PE-Cy7	SK1	Biolegend	#344712
CD4	APC	RPA-T4	Biolegend	#300514
CD3	APC-Cy7	UCHT1	Biolegend	#300426
HLA-DR	Pacific Blue	L243	Biolegend	#307633
CD45	BV510	2D1	Biolegend	#368526
CD11c	PE	S-HCL-3	Biolegend	#371504
CD33	PE-Cy7	WM53	Biolegend	#303434
CD11b	APC	CBRM1/5	Biolegend	#301410
CD14	APC-Cy7	M5E2	Biolegend	#301820
Monoclonal Anti-FMC63 Antibody	PE	Y45	AcroBiosystems	FM3-HPY53-25TESTS
Human BCMA / TNFRSF17 Protein, His Tag	PE	N/A	AcroBiosystems	BCA-HP2H2-25TESTS

Supplemental Table 7: Overview of markers for multiplex cytokine analysis

Marker	Manufacturer	Key event	Marker	Manufacturer	Key event
BAFF	ThermoFisher	Inflammation	vWF-A2	Bio-Techne	Endothelial dysfunction
Fas-L	ThermoFisher	Inflammation	ANG1	Bio-Techne	Endothelial dysfunction
Flt3-Ligand	ThermoFisher	Hematopoietic reserve	ANG2	Bio-Techne	Endothelial dysfunction
G-CSF	ThermoFisher	Hematopoietic reserve	ANG2:ANG1	Bio-Techne	Endothelial dysfunction
GM-CSF	ThermoFisher	Inflammation/ Hematopoietic reserve	E-selectin	Bio-Techne	Endothelial dysfunction
IFN alpha	ThermoFisher	Inflammation	ICAM-1	Bio-Techne	Endothelial dysfunction
IFN gamma	ThermoFisher	Inflammation	MMP-1	Bio-Techne	Endothelial dysfunction
IL-1 alpha	ThermoFisher	Inflammation	P-selectin	Bio-Techne	Endothelial dysfunction
IL-1 beta	ThermoFisher	Inflammation	Thrombomodulin	Bio-Techne	Endothelial dysfunction
IL-2	ThermoFisher	Inflammation	Tie-2	Bio-Techne	Endothelial dysfunction
IL-2R	ThermoFisher	Inflammation	VCAM-1	Bio-Techne	Endothelial dysfunction
IL-3	ThermoFisher	Inflammation			
IL-4	ThermoFisher	Inflammation			
IL-6	ThermoFisher	Inflammation			
IL-6RA	ThermoFisher	Inflammation			
IL-7	ThermoFisher	Inflammation			
IL-8/CXCL8	ThermoFisher	Inflammation			
IL-10	ThermoFisher	Inflammation			
IL-15	ThermoFisher	Inflammation			
M-CSF	ThermoFisher	Hematopoietic reserve			
MCP-1/CCL2	ThermoFisher	Inflammation			
MDC/CCL22	ThermoFisher	Inflammation			
MIG/CXCL9	ThermoFisher	Inflammation			
MIP-1 alpha/CCL3	ThermoFisher	Inflammation			
MIP-1 beta/CCL4	ThermoFisher	Inflammation			
SCF	ThermoFisher	Hematopoietic reserve			
SDF-1 alpha	ThermoFisher	Inflammation			
TNF alpha	ThermoFisher	Inflammation			
Thrombopoietin	ThermoFisher	Hematopoietic reserve			
VEGF-A	ThermoFisher	Endothelial dysfunction			
gp130/IL-6RB	ThermoFisher	Inflammation			

62 **Supplemental Figure 1**

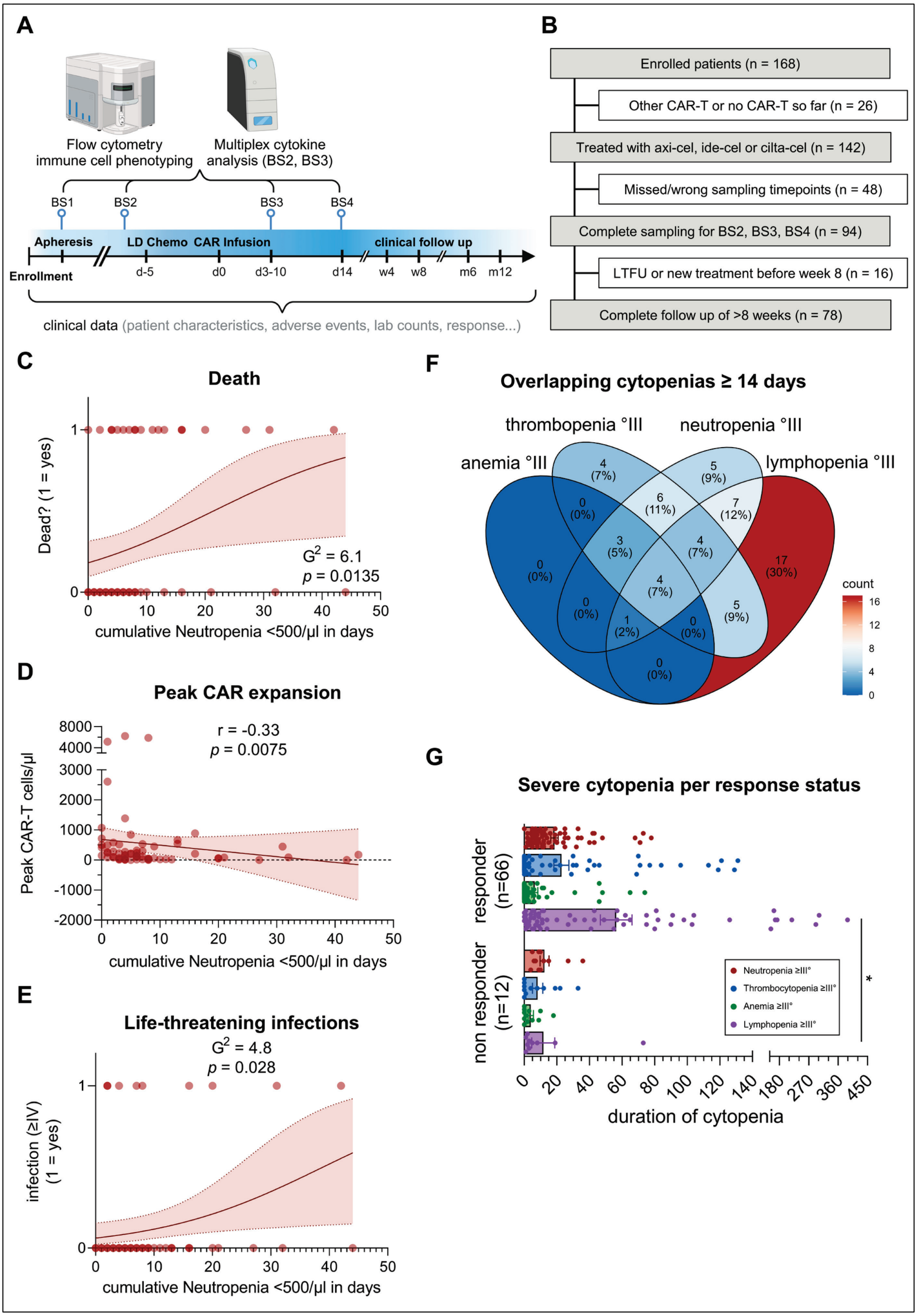

63

64 **Figure S1: Incidence and severity of hematologic toxicities.** (A) Study workflow and sampling
65 timepoints. (B) Patient selection for final analysis. (C) Simple logistic regression analysis comparing

cumulative duration of grade ≥4 neutropenia to occurrence of death. (D) Univariate analysis of the association between peak CAR-T cell expansion and prolonged neutropenia after CAR-T. (E) Simple logistic regression analysis comparing cumulative duration of grade ≥4 neutropenia to occurrence of life-threatening infections (≥ grade 4). For continuous variables the spearman correlation coefficient (r), respective p-value as well as the best-fit line (bold line) and 95% confidence interval (dashed line) as determined by simple linear regression is depicted. Binary variables were analyzed by simple logistic regression and quantified by the likelihood ratio test (G^2). (F) Venn diagram showing the combined intersection (absolute numbers and percentage of the whole) of patients with ≥14 cumulative days of grade ≥3 anemia, thrombocytopenia, neutropenia and/or lymphopenia. (G) Mean cumulative duration of grade ≥3 neutropenia, thrombopenia, anemia and lymphopenia for responders (n=66) vs. non-responders (SD, PD) (n=12). Whiskers indicate standard error of the mean (SEM). Statistical significance was determined by Mann Whitney *U* test (*p<0.05, **p<0.01, ***p<0.001, ****p<0.0001). BS = blood sampling, LD = lymphodepletion, d = day, w = week, m = month, LTFU = lost to follow up.

Supplemental Figure 2

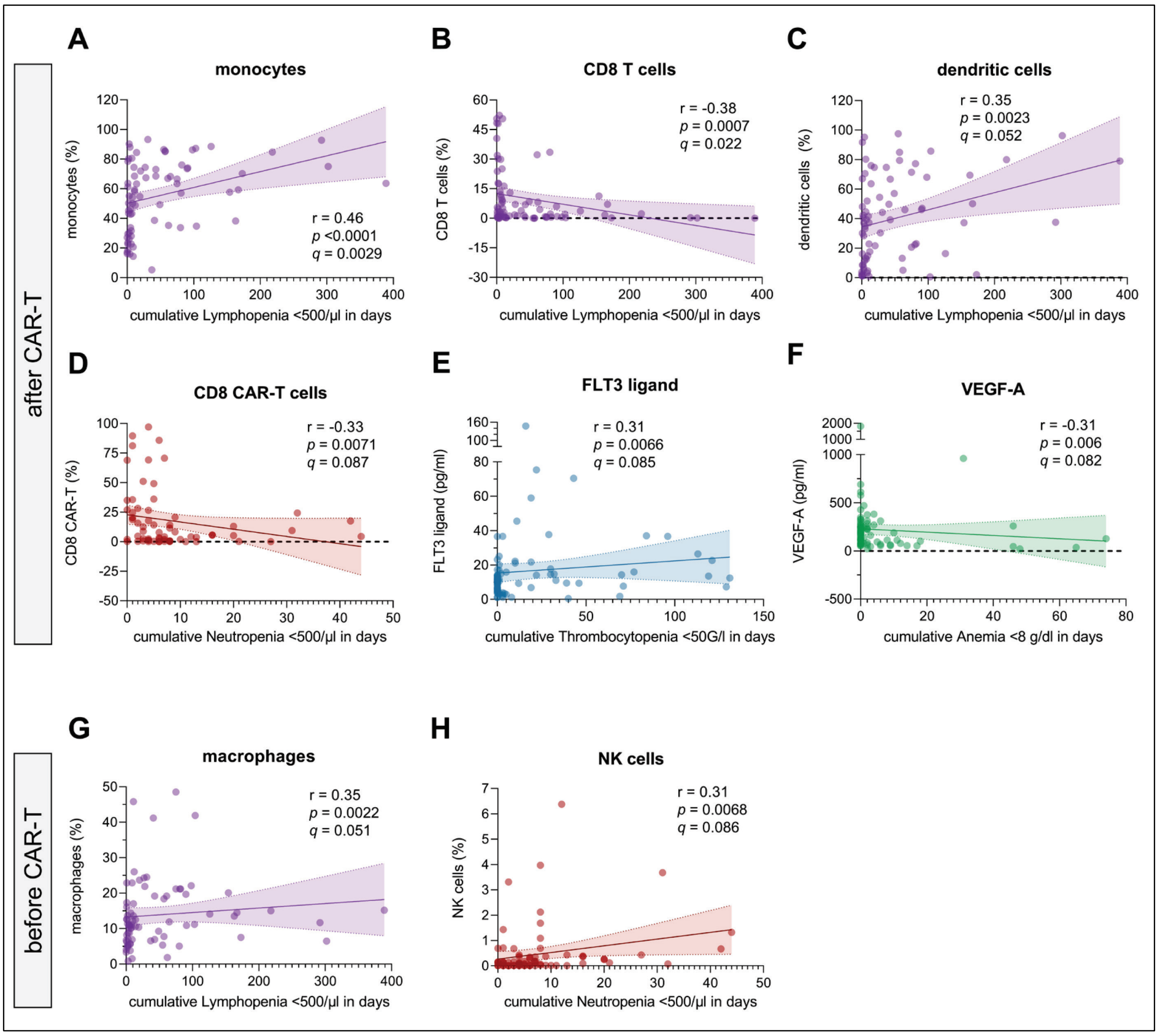

Figure S2: Association of post infusion (A-F) and baseline factors (G-H) with prolonged cytopenia after CAR-T. Univariate analysis of the influence of post infusion monocytes (% of HLA-DR^{+} leukocytes) (A), CD8 T cells (% of CD45^{+} leukocytes) (B) and dendritic cells (% of HLA-DR^{+}CD14^{-} leukocytes) (C) on prolonged lymphopenia (purple), as well as post infusion CD8 CAR-T cells (% of CD8^{+}CD3^{+}CD45^{+} leukocytes) (D), FLT3 ligand (E) and VEGF-A (F) on prolonged neutropenia (red), thrombocytopenia (blue) and anemia (green) after CAR-T. Univariate analysis of the influence of baseline macrophages (% of CD45^{+} leukocytes) on the occurrence of prolonged lymphopenia (purple) after CAR-T (G) and baseline NK cells (% of CD45^{+}CD3^{-} leukocytes) on prolonged neutropenia (red) (H). The Spearman correlation coefficient (r) and respective p- and q-values (after adjustment for multiple testing) are depicted for each marker. Shown are selected markers that fulfilled a q-value cutoff of <0.1.

Supplemental Figure 3

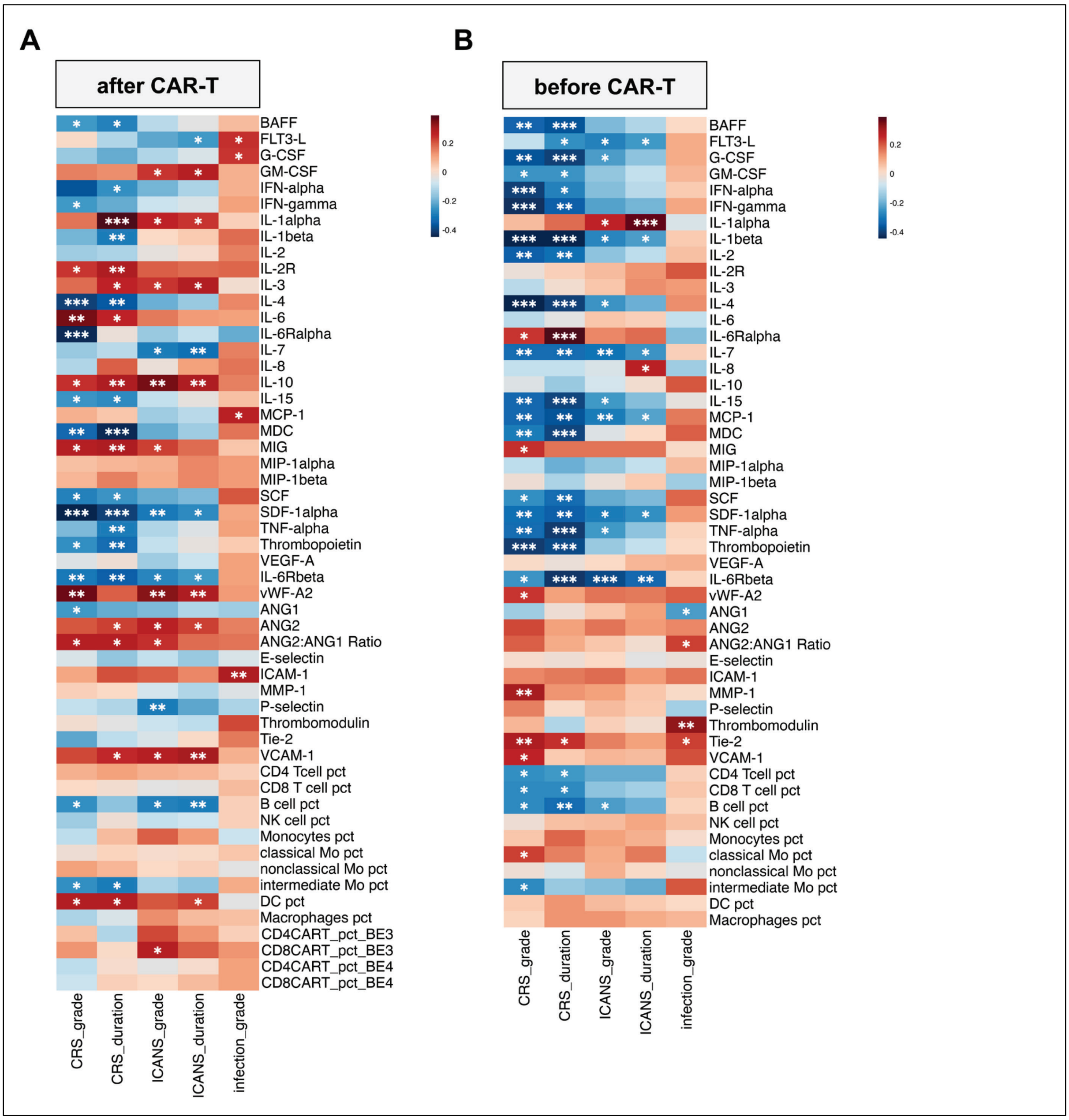

Figure S3: Correlation analysis for other adverse outcomes. Spearman correlation analysis of the influence of cytokines and flow cytometry markers early after CAR-T infusion (range day 3-10) (A) and at baseline (before lymphodepletion) (B) on CRS grade, CRS duration, ICANS grade, ICANS duration and infection grade. The Spearman correlation coefficient (r = scalebar) and the respective p values are depicted in the heatmap with red indicating positive correlation and blue indicating negative correlation. *P < 0.05, **P < 0.01, ***P < 0.001.

Supplemental Figure 4

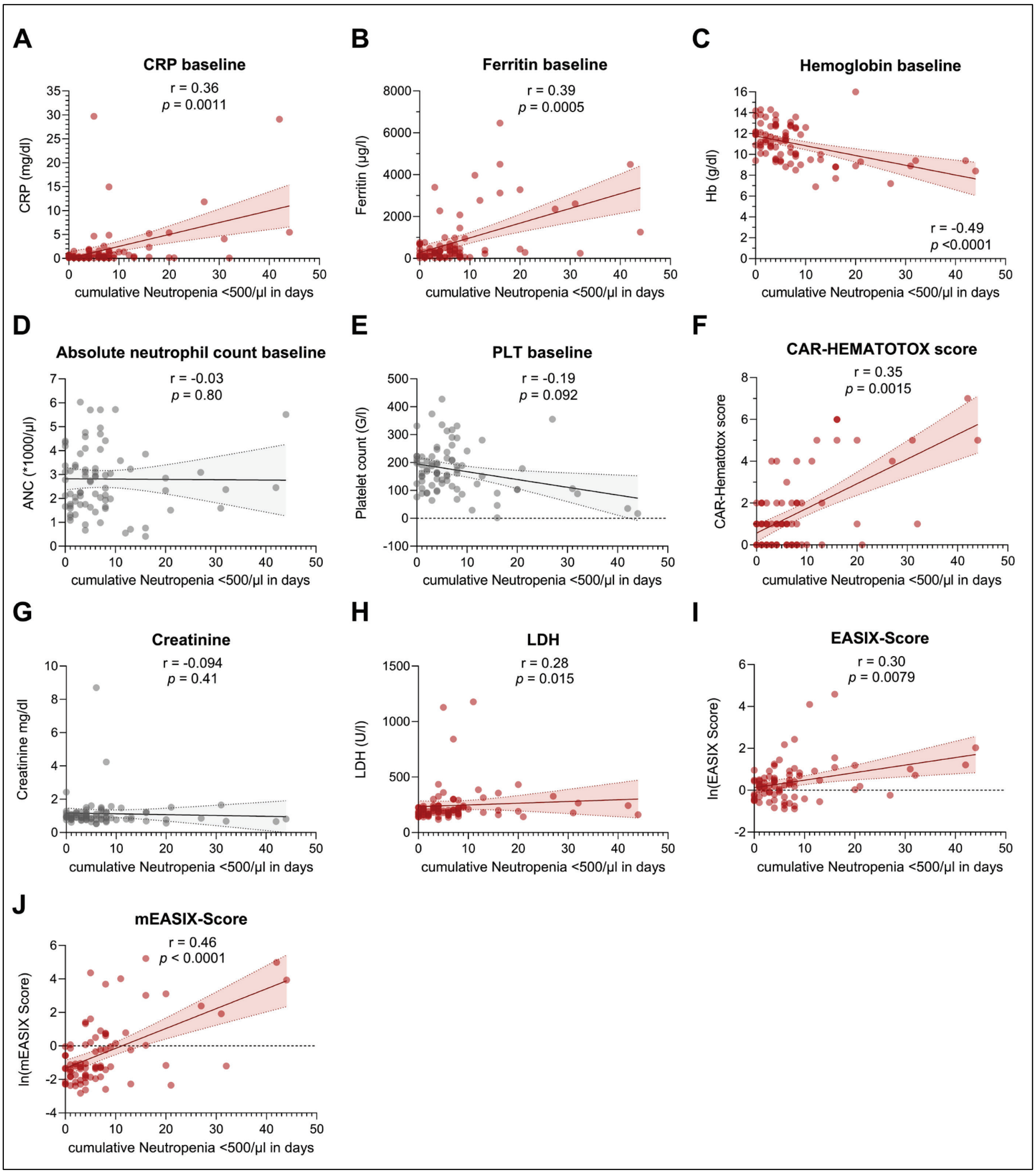

Figure S4: Association between CAR-HEMATOTOX features and prolonged neutropenia after CAR-T. Univariate analysis of the influence of baseline CRP (A), baseline ferritin (B), baseline hemoglobin (C), baseline absolute neutrophil count (ANC) (D), baseline platelet (PLT) count (E) and overall baseline CAR-HEMATOTOX score, as well as baseline creatinine (G), baseline LDH (H) and overall baseline EASIX- (I) and modified-EASIX-Score (J) on the occurrence of prolonged neutropenia after CAR-T. The spearman correlation coefficient (r), respective p-value as well as the best-fit line (bold line) and 95% confidence interval (dashed line) as determined by simple linear regression is depicted.

Supplemental Figure 5

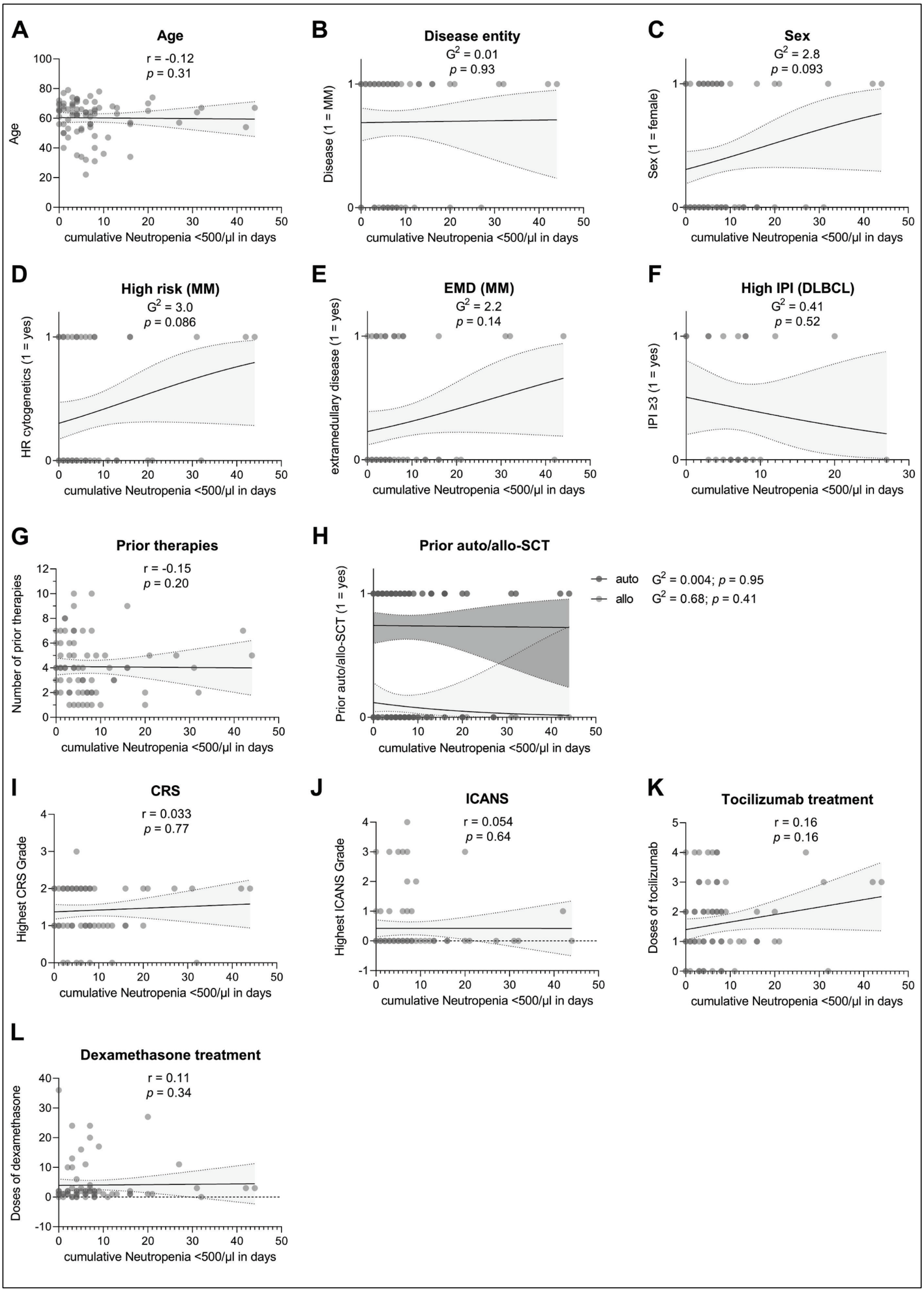

Figure S5: Association between patient characteristics, CRS and ICANS and prolonged neutropenia after CAR-T. Univariate analysis of the influence of age (A), disease entity (B), sex (C), high-risk cytogenetics (MM patients only) (D), extramedullary disease (MM patients only) (E),

international prognostic index (DLBCL patients only) (F), prior lines of therapy (G), prior autologous/allogeneic stem cell transplantation (H), CRS grade (I), ICANS grade (J), tocilizumab (K) and dexamethasone treatment (L) on the occurrence of prolonged neutropenia after CAR-T. For continuous variables the spearman correlation coefficient (r), respective p-value as well as the best-fit line (bold line) and 95% confidence interval (dashed line) as determined by simple linear regression is depicted. Binary variables were analyzed by simple logistic regression and quantified by the likelihood ratio test (G^2).

Supplemental Figure 6

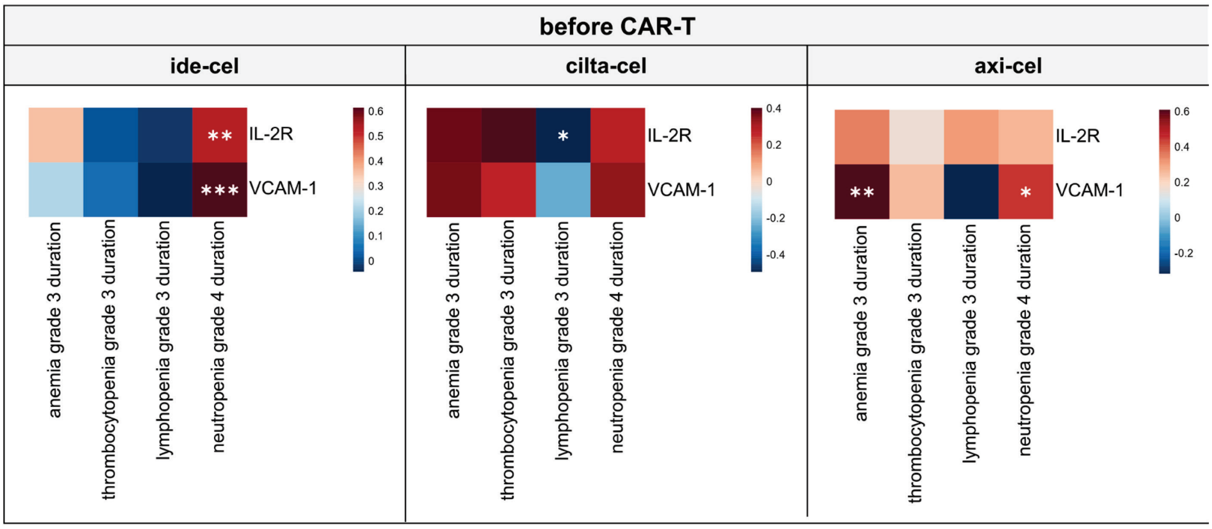

Figure S6: Correlation analysis of sIL-2R and sVCAM-1 per CAR-T product. Spearman correlation analysis of the influence of soluble IL-2R and VCAM-1 at baseline (before lymphodepletion, BS2) on cumulative grade ≥3 anemia, grade ≥3 thrombopenia, grade ≥3 lymphopenia and grade ≥4 neutropenia for ide-cel (left), cilta-cel (middle) and axi-cel (right). The Spearman correlation coefficient (r = scalebar) and the respective p values are depicted in the heatmap with red indicating positive correlation and blue indicating negative correlation. $^*P < 0.05$, $^{**}P < 0.01$, $^{***}P < 0.001$.

Supplemental Figure 7

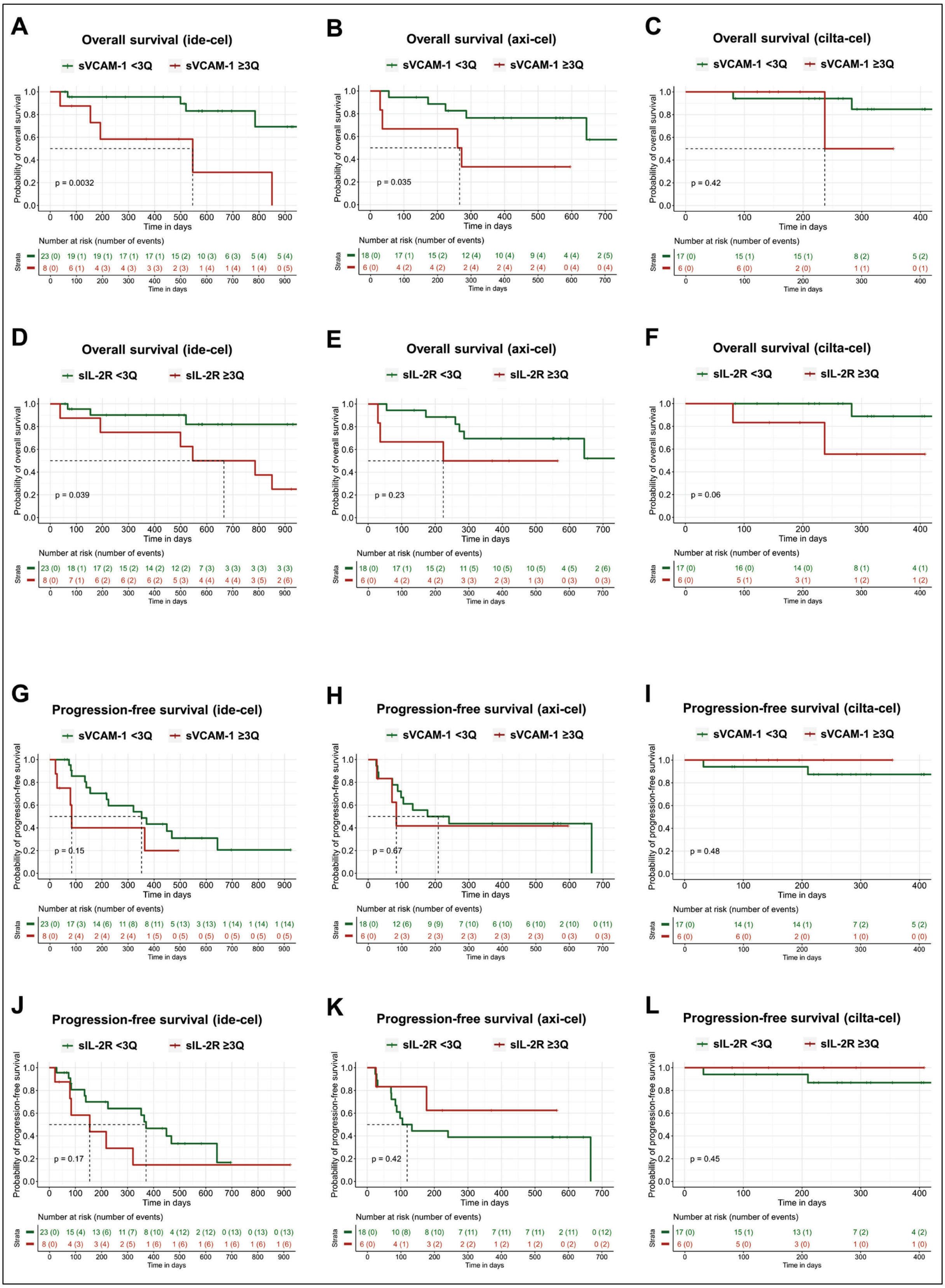

Figure S7: OS and PFS based on baseline sIL-2R and sVCAM-1 for ide-cel, axi-cel and cilta-cel. Kaplan-Meier curves for overall survival (A-F) and progression-free survival (G-L) for ide-cel, axi-cel and cilta-cel with high (red) and low (green) sIL-2R and sVCAM-1 at baseline (before start of lymphodepletion; BS2). Patients with values ≥3 quartile of all patients were characterized as high. The dashed lines indicate the median of overall survival and progression-free survival.

Supplemental Figure 8

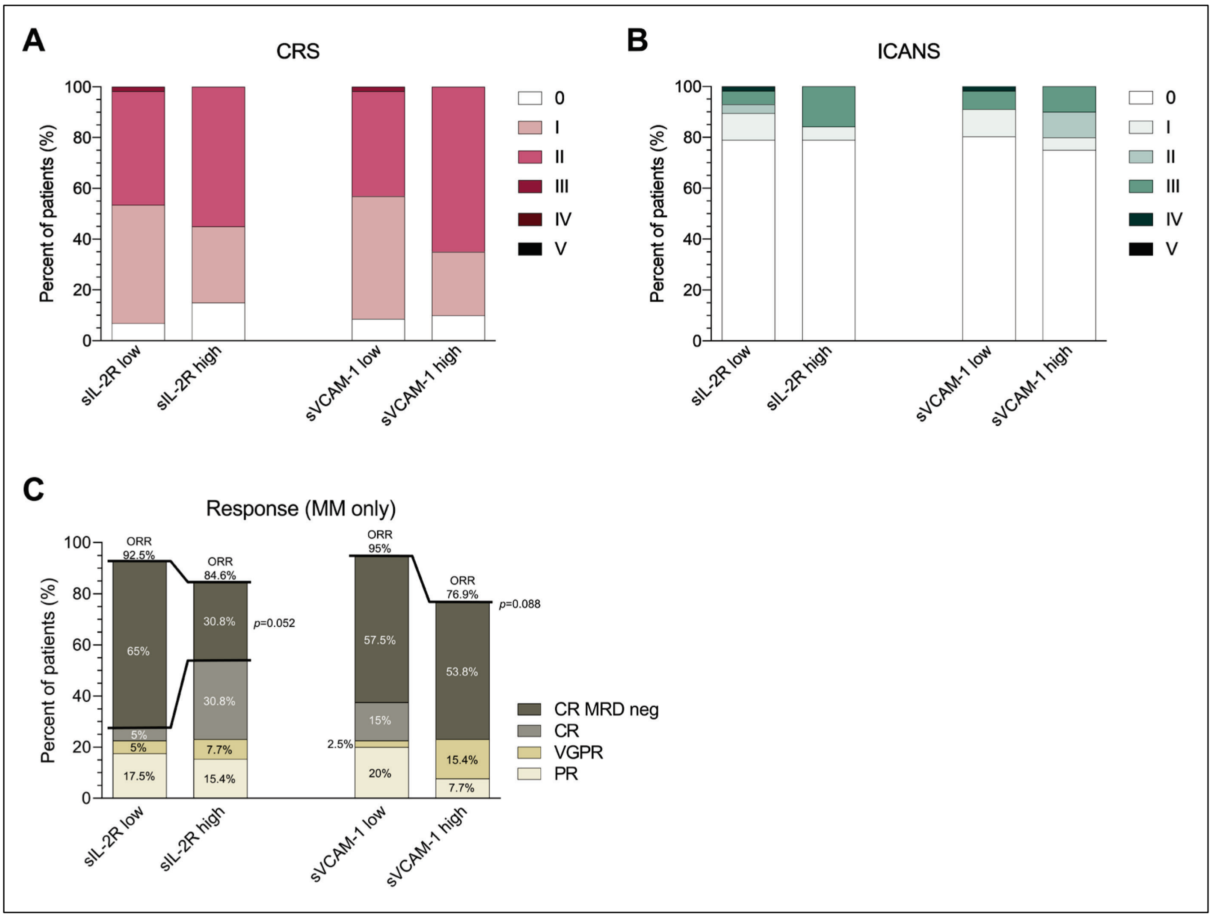

Figure S8: Impact of high baseline sIL-2R and sVCAM-1 on CRS, ICANS and response in MM patients. Distribution of CRS (A) and ICANS grade (B) between patients with high and low sIL-2R or sVCAM-1 at baseline (n=78). (C) Overall response rates of MM patients (n=54) with high and low sIL-2R or VCAM-1 at baseline subdivided by percentage of patients achieving partial remission (PR), very good partial remission (VGPR), complete remission (CR) and MRD negative complete remission (CR MRD neg). Statistical significance was determined by Fisher's exact test (*p<0.05). Patients with values ≥3 quartile of all patients were characterized as high.

Supplemental Figure 9

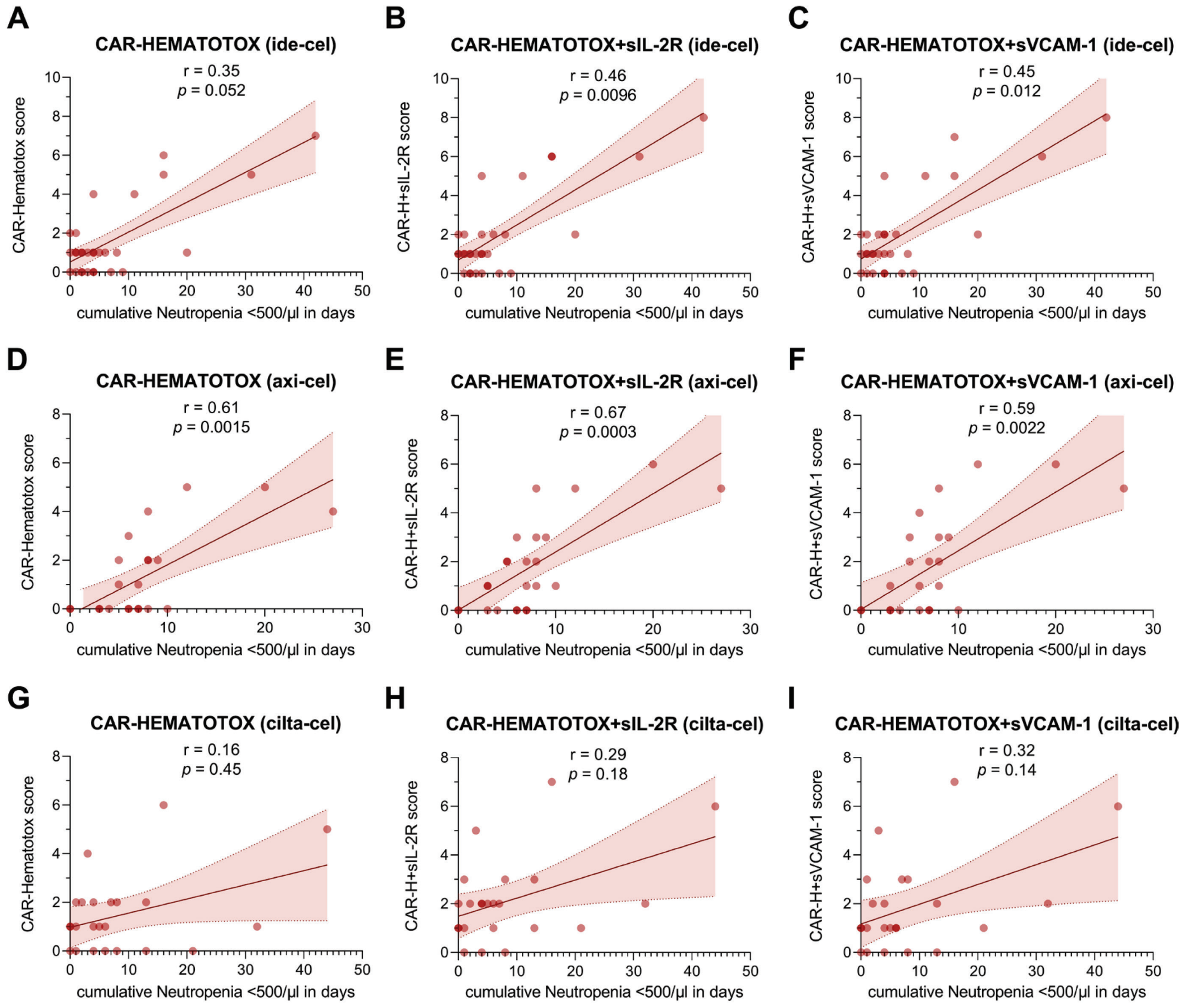

J

**ROC Analysis Neutropenia IV ≥7d**	**Ide-cel**		**Axi-cel**		**Cilta-cel**	
	**AUC**	**p-value**	**AUC**	**p-value**	**AUC**	**p-value**
CAR-HEMATOTOX	0.72	0.058	0.71	0.087	0.60	0.43
CAR-HEMATOTOX + sIL-2R	0.84	0.0033	0.78	0.022	0.64	0.26
CAR-HEMATOTOX + sVCAM-1	0.74	0.035	0.74	0.046	0.67	0.18
EASIX	0.84	0.0031	0.68	0.14	0.73	0.073
EASIX + sIL-2R	0.92	0.0003	0.78	0.019	0.76	0.038
EASIX + sVCAM-1	0.85	0.0023	0.78	0.021	0.78	0.025
mEASIX	0.82	0.0057	0.66	0.17	0.62	0.36
mEASIX + sIL-2R	0.92	0.0003	0.81	0.0091	0.72	0.078
mEASIX + sVCAM-1	0.83	0.0047	0.79	0.018	0.78	0.028

Figure S9: Correlation analysis of CAR-HEMATOTOX score alone and in addition with sIL-2R and sVCAM-1 for axi-cel, cilta-cel and ide-cel patients. Univariate analysis of the influence of CAR hematotox score (n=24 axi-cel, n=23 cilta-cel, n=31 ide-cel), CAR-HEMATOTOX score + baseline sIL-2R and CAR-HEMATOTOX score + baseline sVCAM-1 for axi-cel (A-C) cilta-cel (D-F) and ide-cel (G-I). The spearman correlation coefficient (r), respective p-value as well as the best-fit line (bold line) and 95% confidence interval (dashed line) as determined by simple linear regression is depicted. (J) Tabular summary of ROC analysis of the influence of baseline CAR-HEMATOTOX, EASIX and mEASIX score

alone and plus either baseline sIL-2R or sVCAM-1 on the occurrence of prolonged grade 4 neutropenia ≥7 days for the different CAR-T products ide-cel, axi-cel and cilta-cel. To assess the additive predictive value of our biomarkers, ROC analyses were performed for sIL-2R and sVCAM-1 for each CAR-T product to determine optimal cutoffs maximizing the Youden index. For the CAR-HEMATOTOX score, which is a categorized composite model, our biomarkers were added by assigning one additional point if the respective marker exceeded its threshold and were compared to the regular CAR-HEMATOTOX scoring system in ROC analysis. For EASIX and mEASIX, which are continuous scores, we first derived CAR-T product-specific cutoffs using ROC analysis and then binarized the scores. Composite models were constructed by combining the binarized EASIX/mEASIX scores with our biomarkers, and ROC analysis was used to compare the predictive performance of the binarized original and combined models.

Supplemental Figure 10

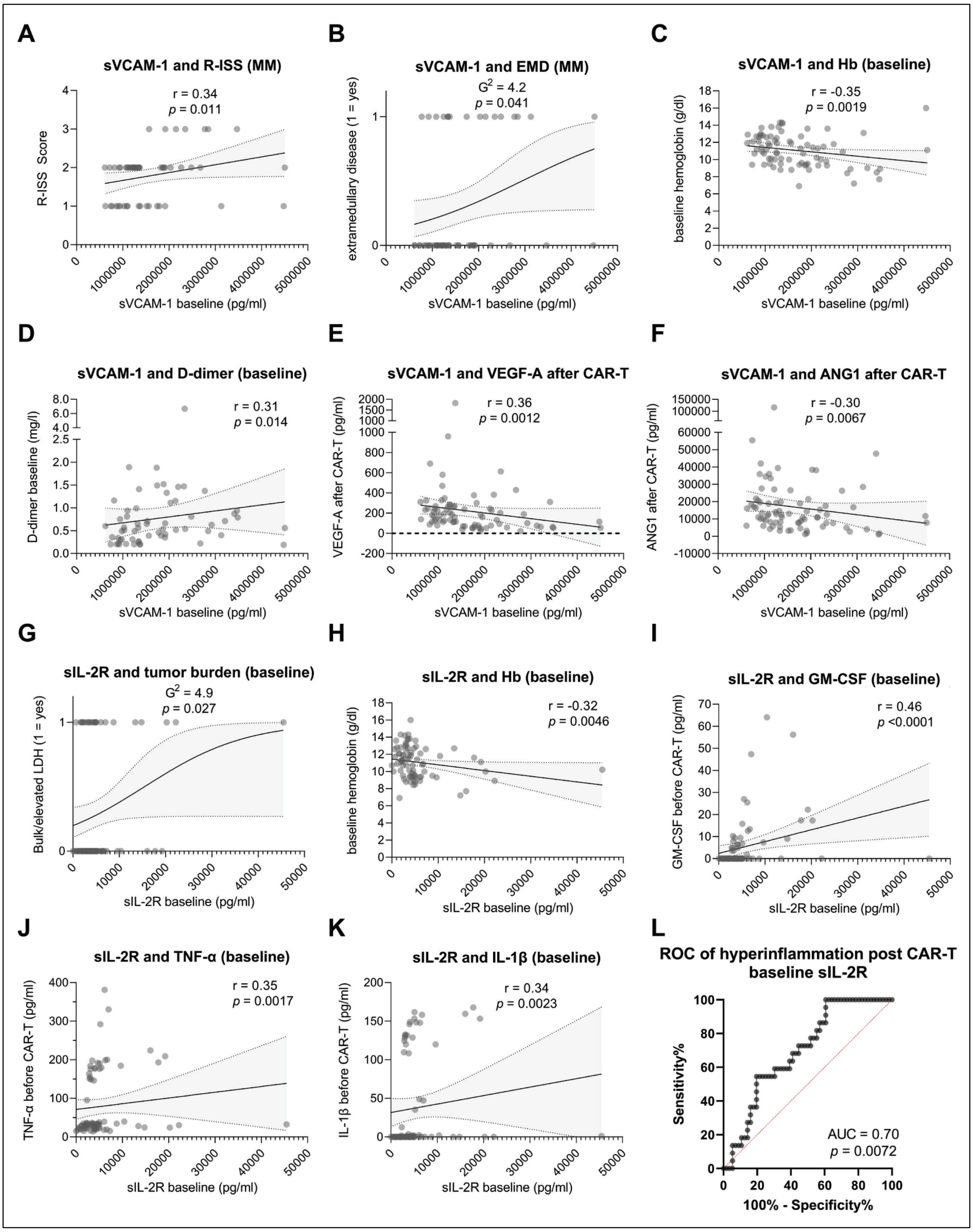

Figure S10: Association between baseline sIL-2R and sVCAM-1 and other factors. Univariate analysis of the association between baseline sVCAM-1 and baseline R-ISS (A), extramedullary disease (B), baseline hemoglobin (C), baseline d-dimer (D) as well as VEGF-A (E) and ANG1 (F) after CAR-T. Univariate analysis of the association between baseline sIL-2R and baseline tumor burden (G), baseline hemoglobin (H), baseline GM-CSF (I), baseline TNF-α (J) and baseline IL-1β (K). (L) Receiver operating characteristic (ROC) curves of the influence of baseline sIL-2R on a hyperinflammatory response after CAR-T, characterized by at least two inflammatory cytokines (GM-CSF, IL-1β, TNF-α or IFN-γ) above the third quartile after CAR-T. For correlation analysis the spearman correlation coefficient (r), respective p-value as well as the best-fit line (bold line) and 95% confidence interval (dashed line) as determined

by simple linear regression is depicted. For ROC curves the respective area under the curve (AUC) and p-value are depicted.

Supplemental Figure 11

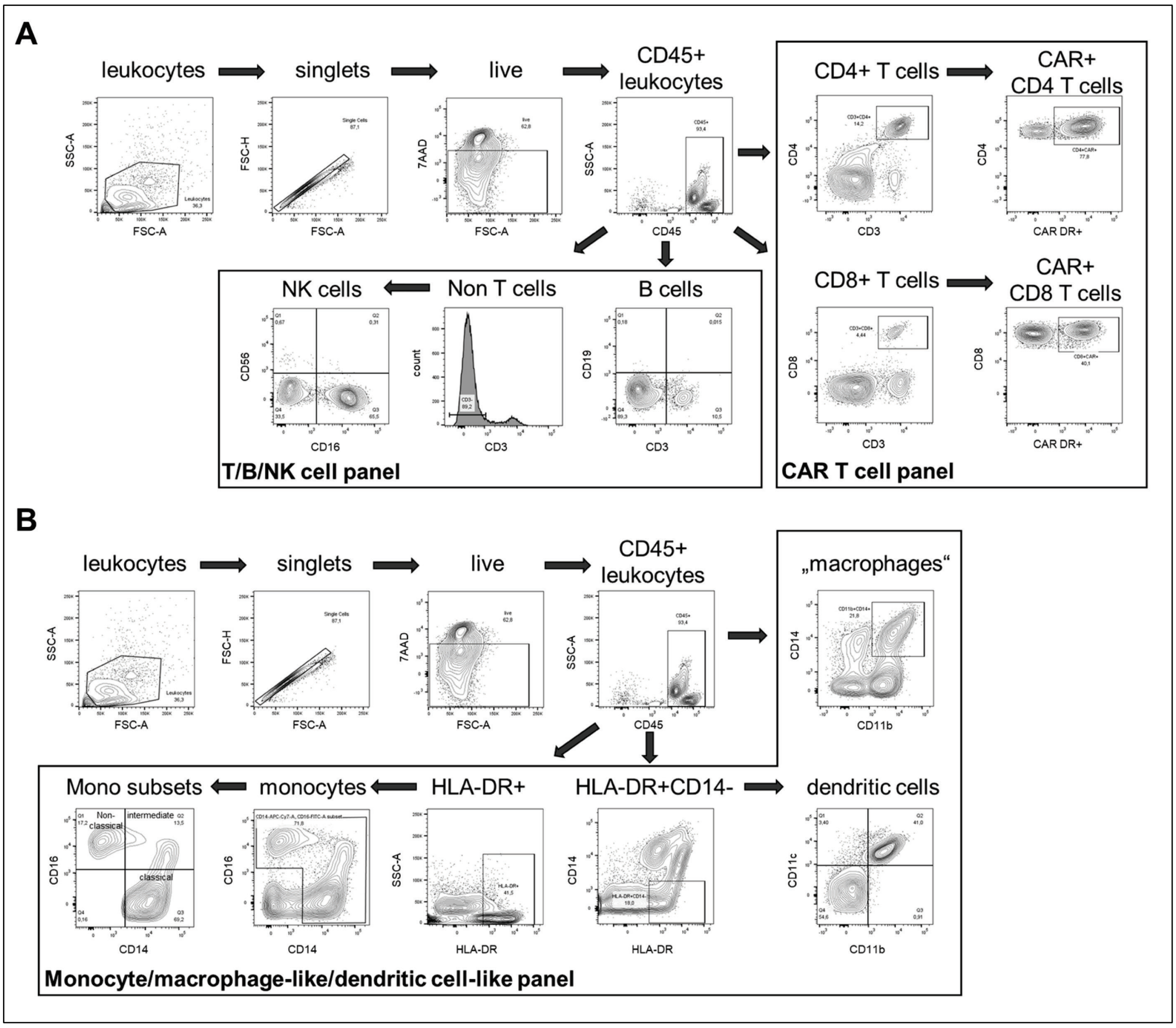

Figure S11: Flow cytometry gating strategy. (A) Gating strategy for T/B/NK cell panel and CAR-T cell panel. (B) Gating strategy for monocyte/macrophage/dendritic-cell-like panel.
